# Phytochemical Modulation of Oxidative Stress–Inflammation Coupling in Cardiovascular Disease: From Nrf2 and NADPH Oxidases to NLRP3 and Endothelial Dysfunction

**DOI:** 10.3390/antiox15101222

**Published:** 2026-09-22

**Authors:** Siwon Baek, Minseung Kang, Giuseppe Maria Raffa, Calogera Pisano, Vincenzo Nuzzi, Mariusz Kowalewski, Piotr Suwalski, Tomasz Urbanowicz

**Affiliations:** 1 College of Medicine, Inje University, 75 Bokji-ro, Busanjin-gu, Busan 47392, Republic of Korea; 2 College of Medicine, Korea University, 73 Goryeodae-ro, Seongbuk-gu, Seoul 02841, Republic of Korea; 3 Department of Precision Medicine in Medical Surgical and Critical Area (Me.Pre.C.C.), University of Palermo, 90134 Palermo, Italy; 4 IRCCS ISMETT, Department of Research, Via Tricomi 5, 90127 Palermo, Italy; 5 Thoracic Research Center, Collegium Medicum, Nicolaus Copernicus University, 85-094 Bydgoszcz, Poland; 6 Department of Cardiac Surgery, Center of Postgraduate Medical Education, Central Clinical Hospital of the Ministry of Interior, 02-507 Warszawa, Poland; 7 Cardiac Surgery and Transplantology Department, Poznan University of Medical Sciences, ½ Długa, 61-848 Poznań, Poland

**Keywords:** cardiovascular disease, Nrf2, NADPH oxidase, NLRP3 inflammasome, endothelial dysfunction, phytochemicals, oxidative stress, bioavailability, gut microbiota, precision phytotherapy

## Abstract

Oxidative stress and inflammation are increasingly recognized as reciprocally reinforcing components of cardiovascular disease rather than independent pathological processes. Their interaction is organized through a network that includes mitochondrial dysfunction, NADPH oxidases (NOX), redox-sensitive NF-κB signaling, NLRP3 inflammasome activation, endothelial nitric oxide synthase (eNOS) uncoupling, and loss of nitric oxide bioavailability. Within this framework, phytochemicals are more appropriately viewed as modulators of endogenous redox–inflammatory signaling than as simple radical scavengers. This review critically examines the evidence linking major phytochemical classes to the Nrf2–NOX–ROS–NF-κB/NLRP3–eNOS/NO axis and evaluates how these mechanisms translate across cardiovascular phenotypes, with emphasis on mechanistic hierarchy, as simultaneous pathway changes may reflect propagation from limited upstream targets rather than direct multi-target activity. The review addresses disease-stage dependence, context-specific roles of Nrf2 and NOX isoforms, mitochondrial–NOX crosstalk, pyroptosis, endothelial dysfunction, and the translational importance of pharmacokinetics, formulation, microbial biotransformation, and drug interactions. Current evidence supports a shift from generalized antioxidant supplementation toward mechanism-matched intervention in biologically selected patients, with the greatest credibility achieved when the active molecular species, dominant mechanism, cardiovascular phenotype, disease stage, clinically relevant exposure, molecular target engagement, functional benefit, and clinical outcomes are evaluated within an integrated framework.

## 1. Introduction

### 1.1. Cardiovascular Disease and the Need for New Therapeutic Approaches

Cardiovascular disease (CVD) remains the leading cause of death worldwide [1]. Despite major advances in prevention, drug therapy, and interventional treatment, substantial cardiovascular risk persists in many treated patients [2]. This has increased interest in biological processes that are not fully captured by conventional risk assessment. Oxidative stress and inflammation are among the most important of these processes because they contribute to vascular injury, atherosclerosis, myocardial remodeling, and heart failure [2].

Oxidative stress and inflammation are not separate processes in cardiovascular disease. Reactive oxygen species (ROS) are required for normal cell signaling, vascular homeostasis, metabolic adaptation, and host defense [3]. Their effects depend on where, how much, and for how long they are produced. In cardiovascular disease, excessive ROS from NOX enzymes, damaged mitochondria, and uncoupled endothelial nitric oxide synthase (eNOS) oxidize lipids and proteins, reduce nitric oxide (NO) bioavailability, and activate inflammatory pathways [4,5]. Inflammation then increases ROS production through cytokine signaling, leukocyte activation, and mitochondrial dysfunction [4,5]. Together, these processes form a self-reinforcing redox–inflammatory network.

Several molecular systems connect the redox and inflammatory components of this network. Nrf2 coordinates endogenous defenses against oxidative and electrophilic stress. NOX enzymes generate localized ROS signals in cardiovascular tissues in a regulated and context-dependent manner [6]. Redox-sensitive activation of NF-κB and the NLRP3 inflammasome links disrupted redox balance to cytokine release, innate immune activation, and pyroptotic cell death [7]. At the vascular endothelium, oxidative injury, inflammation, and eNOS uncoupling reduce NO availability and promote leukocyte adhesion, vasoconstriction, and thrombosis [6]. Endothelial dysfunction is therefore a functional result of disturbed redox–inflammatory homeostasis.

### 1.2. Limitations of Conventional Antioxidant Approaches

Conventional antioxidant strategies have produced disappointing cardiovascular results [8,9,10,11]. One reason is that ROS are not only damaging molecules; at low and transient levels, they also act as signals. During ischemic preconditioning, for example, a brief increase in ROS activates protective pathways that help the myocardium tolerate a later ischemic insult [12]. By contrast, sustained or excessive ROS production promotes endothelial dysfunction and tissue injury [3,4]. Effective redox-directed interventions should therefore control harmful ROS sources while preserving physiological redox signaling.

### 1.3. Why Phytochemicals Require a Network-Based Interpretation

Against this background, phytochemicals have attracted interest as potential regulators of cardiovascular redox and inflammatory pathways. Reported actions include activation of Nrf2-dependent defenses, attenuation of NF-κB-associated inflammatory signaling, and preservation of endothelial NO signaling [6,13,14]. Such effects may arise from the regulation of signaling pathways and gene expression rather than from direct chemical scavenging of ROS alone [14,15]. Phytochemicals may therefore act on pathways linking redox regulation and inflammatory signaling instead of functioning simply as nonspecific antioxidants.

However, the broad range of reported phytochemical effects creates an important problem of mechanistic interpretation. Changes in Nrf2, NOX, NF-κB, NLRP3, mitochondrial ROS, or eNOS after treatment do not by themselves establish that a compound acts directly on each target [13,14,15]. Modulation of a limited number of upstream processes may produce several downstream changes. The simultaneous measurement of multiple pathway markers should therefore not automatically be interpreted as evidence of direct multi-target activity.

Mechanistic interpretation is further complicated by the context-dependent functions of the pathways being measured. The Keap1–Nrf2 pathway often protects cells from oxidative stress, but prolonged or excessive activation may not always be beneficial [16]. NOX-derived ROS can also be protective or harmful depending on their source, amount, and duration [17]. A phytochemical-induced increase or decrease in any pathway cannot therefore be classified as beneficial without considering its magnitude, duration, cellular location, disease stage, and biological context.

Even a biologically favorable mechanism has limited translational value if the required exposure cannot be achieved in humans. Many cell-culture studies use concentrations far above those reached after oral dosing [15]. Culture conditions can also alter redox behavior, and some phytochemicals may become pro-oxidant at high concentrations [15]. In humans, exposure depends on absorption, intestinal and hepatic metabolism, formulation, tissue distribution, and gut microbial conversion. Cardiovascular tissues may therefore encounter metabolites that differ substantially from the parent compound tested in vitro [15].

Clinical studies suggest that some phytochemicals may improve markers such as endothelial function and blood pressure [18]. However, findings vary across studies, and improvements in these markers do not establish reductions in major cardiovascular events [18,19]. The key questions are whether a biologically relevant exposure can be achieved in humans and whether the proposed target is engaged at that exposure. Studies must then show that target engagement produces measurable cardiovascular benefit.

### 1.4. Objectives of the Review

This review examines these questions through the Nrf2-NOX-ROS-NF-κB/NLRP3-eNOS/NO axis. Instead of cataloguing every antioxidant compound, it focuses on points where redox regulation and inflammatory signaling intersect and on evidence that phytochemicals can modify these interactions. The review then considers these mechanisms in atherosclerosis, hypertension, ischemia–reperfusion injury, heart failure, diabetes-associated cardiovascular disease, atrial fibrillation, and abdominal aortic aneurysm.

A second objective is to separate mechanistic promise from clinical plausibility. We therefore consider pharmacokinetics, bioavailability, formulation, gut microbiota-dependent metabolism, and interactions with established cardiovascular therapies alongside molecular mechanisms. This translational perspective supports a shift from broad antioxidant supplementation toward mechanism-informed, biomarker-guided precision phytotherapy.

### 1.5. Literature Search and Scope

The expanded narrative review was informed by a structured PubMed search updated through August 2026. Search concepts covered phytochemicals and major compound classes; the Nrf2/Keap1, NADPH oxidase, mitochondrial ROS, NF-κB, NLRP3, pyroptosis, and eNOS/NO pathways; cardiovascular phenotypes; human pharmacokinetics; formulation; microbial biotransformation; and drug interactions. Recent evidence was prioritized, while foundational studies were retained when they defined mechanisms or pharmacokinetic constraints that remain central to the field.

Evidence was interpreted according to study level and the claim being made. Cell and animal studies were used to assess biological plausibility and pathway dependence. Human pharmacokinetic studies were used to judge achievable exposure. Randomized trials and meta-analyses were prioritized for vascular and cardiometabolic effects, whereas cardiovascular outcome trials were used to assess clinical validation of inflammatory or antioxidant concepts. Because this is a narrative rather than a systematic review, the search was designed for critical mechanistic synthesis rather than exhaustive enumeration of studies or pooled effect estimation. Stroke and peripheral artery disease were not developed as separate phenotype sections because the review focused on vascular and myocardial settings, where the most direct evidence for phytochemicals was developed.

## 2. Cardiovascular Redox–Inflammatory Framework

Oxidative stress and inflammation are closely linked in cardiovascular disease [4,5,20,21,22]. Their interaction involves more than the simultaneous accumulation of reactive oxygen species (ROS) and inflammatory mediators. Redox-sensitive transcription, mitochondrial dysfunction, NOX activity, eNOS uncoupling, innate immune signaling, and cytokine-driven ROS production form an interconnected system. A change in one component can alter several others. This two-way interaction is especially important in the vascular wall, where endothelial cells, vascular smooth-muscle cells, and immune cells generate distinct but interacting redox and inflammatory signals. This section provides a concise map of the network before the later sections examine each pathway and its phytochemical modulation in greater detail.

### 2.1. ROS as Physiological Signaling Molecules

Reactive oxygen species (ROS) are oxygen-derived reactive molecules produced during normal cellular metabolism and in response to cellular stress [23,24]. They include chemically distinct species such as superoxide and hydrogen peroxide. ROS are not inherently harmful; their biological effects depend on where, how much, and for how long they are produced [23,24]. Cells maintain this balance by regulating ROS production within specific cellular compartments and by using antioxidant enzymes, including superoxide dismutases, catalase, glutathione peroxidases, and peroxiredoxins, to limit excessive ROS accumulation [23,24]. Nrf2 further supports redox control by increasing the expression of antioxidant and cytoprotective genes during oxidative stress [13,14]. Its detailed regulation is discussed in Section 3.1. At low, controlled levels, ROS act as signaling molecules that regulate vascular tone, cell growth, metabolic adaptation, and host defense [23,24,25,26]. These signals generally involve the reversible oxidation of redox-sensitive proteins rather than nonspecific molecular damage [23,25].

This process is particularly relevant to ischemia–reperfusion, in which rapid metabolic changes during reperfusion produce a large mitochondrial oxidative burst [27,28,29]. Persistent mitochondrial dysfunction can then contribute to inflammation, cell death, and adverse ventricular remodeling [28,30,31]. During ischemic preconditioning, however, transient mitochondrial ROS act as signals that help trigger cardioprotective pathways [12,28,29]. The effect of mitochondrial ROS therefore depends on their magnitude, timing, and persistence.

### 2.2. Transition from Redox Signaling to Oxidative Stress

Pathological oxidative stress develops when the balance between ROS production and removal is disrupted. Excessive or prolonged ROS production, insufficient antioxidant defenses, or ROS generation in inappropriate cellular compartments can damage proteins, lipids, and nucleic acids [24,32]. In endothelial cells, superoxide reacts with nitric oxide (NO) to form peroxynitrite, thereby reducing NO bioavailability while increasing oxidative and nitrosative stress [32,33]. The resulting loss of NO impairs vasodilation and promotes platelet activation, leukocyte adhesion, vascular smooth-muscle-cell growth, and inflammatory signaling [33,34].

Peroxynitrite also oxidizes tetrahydrobiopterin and promotes eNOS uncoupling [34,35,36]. Once uncoupled, eNOS produces superoxide instead of NO. Thus, an enzyme that normally supports vascular homeostasis becomes an additional source of oxidative stress, further reducing NO bioactivity [34,35,36].

These findings show that cardiovascular oxidative stress is not simply a global rise in ROS. It reflects a loss of location-specific redox control and an imbalance between ROS production and removal [23,24,37]. Nonspecific ROS suppression may therefore disrupt protective signaling. Current strategies instead aim to control defined ROS sources and their downstream effects [37,38].

### 2.3. Major Cardiovascular Sources of ROS

Mitochondria are central regulators of myocardial and vascular redox homeostasis. In addition to their primary role in oxidative phosphorylation, they integrate metabolic state, calcium handling, redox signaling, and cell-death pathways. Mitochondrial ROS generation is consequently linked closely to cellular function and is not, in itself, evidence of mitochondrial injury. The pathological transition occurs when mitochondrial ROS production becomes excessive or sustained and begins to impair the organelle from which it originates [27].

Several conditions central to cardiovascular disease—including hyperglycemia, lipid excess, ischemia–reperfusion, and inflammatory cytokine exposure—can disturb mitochondrial electron transport and increase electron leakage [25,27,32,39]. The resulting oxidative environment can damage respiratory-chain components, mitochondrial proteins, lipids, and mitochondrial DNA, thereby further impairing electron transport and establishing a feed-forward mechanism of mitochondrial dysfunction [32,40].

One clear manifestation of this amplification is ROS-induced ROS release (RIRR). Oxidative stress can increase mitochondrial membrane permeability and promote opening of the mitochondrial permeability transition pore, producing abrupt changes in membrane potential and ROS generation. ROS released from affected mitochondria can subsequently alter the function of neighboring mitochondria, allowing an initially localized disturbance to propagate through the mitochondrial network. RIRR therefore provides a mechanistic link between discrete mitochondrial injury and broader cellular oxidative stress [41].

NADPH oxidases differ from most other cellular ROS sources because regulated ROS generation is their primary enzymatic function, rather than a secondary consequence of metabolism or enzyme dysfunction. NOX enzymes function as dedicated redox-signaling systems in cardiovascular cells. Receptor activation, shear stress, mechanical stretch, and calcium signals can regulate specific NOX isoforms through changes in enzyme expression, assembly of regulatory subunits, phosphorylation, and subcellular localization [42,43]. The resulting ROS are generated within defined cellular microdomains, where they reversibly modify nearby redox-sensitive proteins and help coordinate endothelial adaptation, vascular smooth-muscle-cell responses, and myocardial responses to mechanical or metabolic stress [42,43,44].

The biological effect of NOX activity depends on the isoform and cell type, as well as the ROS, location, amount, and duration of production [42,44]. Transient and spatially restricted activity supports physiological signaling, whereas loss of isoform- and compartment-specific control can shift NOX activity toward redox imbalance [44,45,46]. The distinct roles of cardiovascular NOX isoforms and their interactions with mitochondria and eNOS are examined in Section 3.2.

### 2.4. Formation of the Redox–Inflammatory Feedback Cycle

The intersection between oxidative stress and inflammation is mediated partly by redox-sensitive transcriptional and innate immune pathways. NF-κB is a central component of this interface. Changes in the cellular redox state influence NF-κB signaling, while NF-κB regulates the expression of cytokines, chemokines, and adhesion molecules. Redox-sensitive NF-κB activation therefore converts disturbed redox homeostasis into pro-inflammatory gene expression [20,21,22].

Activated inflammasome signaling can, in turn, alter the local redox environment. Cytokine release, leukocyte recruitment, and inflammatory cell death increase metabolic and ROS-generating activity in affected tissues. Inflammation therefore does not simply occur downstream of oxidative stress; it can maintain and amplify the redox disturbance that contributed to its initiation. This reciprocal reinforcement provides the molecular basis for persistent redox–inflammatory coupling in cardiovascular tissues [22,47].

### 2.5. From Cellular Signaling to Cardiovascular Remodeling

The consequences of sustained redox–inflammatory coupling become evident when an initially reversible cellular response is maintained over time. Acute ROS and inflammatory signaling participate in normal adaptation to tissue stress and injury. Persistent activation, by contrast, changes endothelial phenotype, promotes leukocyte recruitment, alters vascular smooth-muscle-cell behavior, and contributes to extracellular-matrix remodeling and fibrosis [22,48].

The vascular endothelium provides an early mechanistic example of this transition. Classical experiments demonstrated that cytokine-induced expression of vascular cell adhesion molecule-1 (VCAM-1) in human endothelial cells is regulated through an antioxidant-sensitive, NF-κB-associated transcriptional mechanism [49]. These observations established an important connection between cellular redox state and endothelial inflammatory activation. Increased expression of VCAM-1 and other adhesion molecules facilitates leukocyte adhesion and migration into the vascular wall, converting intracellular redox signaling into a cellular inflammatory response [22,48,49].

Once inflammatory cells accumulate within the vessel wall, additional sources of ROS, proteases, cytokines, and lipid mediators are introduced into the local environment. Vascular smooth-muscle cells undergo phenotypic changes that influence migration, proliferation, matrix synthesis, and plaque architecture. Endothelial NO signaling deteriorates further, while oxidative modification of lipids and proteins provides additional inflammatory stimuli. The resulting interactions contribute to the progression of atherosclerosis, vascular stiffening, endothelial dysfunction, and a prothrombotic vascular phenotype [22,47,48].

Comparable processes operate within the myocardium. Persistent mitochondrial dysfunction and inflammatory signaling promote cardiomyocyte injury and death, while cytokine- and ROS-dependent activation of fibroblasts contributes to extracellular-matrix deposition and structural remodeling. Thus, although the cellular participants differ among atherosclerosis, ischemic injury, hypertension, and heart failure, the persistence of mutually reinforcing redox and inflammatory signaling represents a recurring feature of cardiovascular remodeling [30,31,50].

## 3. Core Redox–Inflammatory Targets of Phytochemical Cardioprotection

Building on Section 2, this section examines the major molecular systems that regulate cardiovascular redox–inflammatory signaling in detail: Keap1–Nrf2–ARE, NOX, NF-κB–NLRP3–pyroptosis, and eNOS/NO. We distinguish physiological regulation from disease-associated dysregulation and evaluate how phytochemicals modify these interconnected pathways.

### 3.1. The Keap1–Nrf2–ARE Pathway

Nuclear factor erythroid 2-related factor 2 (Nrf2) is a major transcriptional regulator of cellular adaptation to oxidative and electrophilic stress [51,52]. In cardiovascular cells, Nrf2 regulates not only antioxidant enzyme expression but also glutathione and thioredoxin metabolism, xenobiotic detoxification, intermediary metabolism, mitochondrial homeostasis, and cellular stress resistance [51,52,53,54,55,56,57,58,59]. Its interaction with inflammatory signaling pathways further links the adaptive stress response to vascular and myocardial injury [20]. Nrf2 therefore connects the sensing of redox disturbances to a coordinated cellular response to cardiovascular stress [51].

#### 3.1.1. Basal Regulation of Nrf2

Under basal conditions, Nrf2 activity is controlled predominantly by Kelch-like ECH-associated protein 1 (Keap1), which acts as a substrate adaptor for a Cullin 3-based E3 ubiquitin–ligase complex [52,60]. Keap1-dependent ubiquitination targets Nrf2 for continuous proteasomal degradation and maintains a short intracellular half-life [60]. Continuous turnover allows Nrf2 levels to change rapidly when the intracellular redox or electrophilic environment is altered [52].

Keap1 contains reactive cysteine residues that act as sensors of oxidative and electrophilic stress [52,60]. Modification of these residues impairs efficient Keap1-dependent ubiquitination, allowing newly synthesized Nrf2 to accumulate and translocate to the nucleus [52,60]. Nuclear Nrf2 heterodimerizes with small Maf proteins and binds antioxidant response elements (AREs) in the regulatory regions of target genes, initiating a coordinated transcriptional response that promotes cellular adaptation and recovery [52].

The Keap1–Nrf2 system is therefore better understood as an adaptive stress-response pathway than as a conventional antioxidant pathway [15,51]. Nrf2 does not neutralize ROS directly. Instead, it increases the cellular capacity to metabolize reactive intermediates, maintain redox balance, and withstand subsequent stress [51,52]. Many phytochemicals traditionally described as antioxidants may influence this pathway through electrophilic or redox signaling rather than through direct radical scavenging alone [15].

Nrf2 activity can also be regulated through Keap1-independent signaling. Glycogen synthase kinase 3β (GSK-3β) promotes β-TrCP-dependent ubiquitination and degradation of Nrf2, whereas phosphoinositide 3-kinase/Akt signaling inhibits GSK-3β and can increase Nrf2 stability and nuclear activity. This mechanism helps explain how growth-factor signaling and some phytochemicals can enhance Nrf2 activity without directly modifying Keap1 [61,62].

#### 3.1.2. Nrf2-Dependent Cytoprotective Programs

Nrf2 regulates a broad range of cytoprotective genes. It supports glutathione synthesis by regulating glutamate–cysteine ligase and promotes glutathione-dependent detoxification through glutathione S-transferases [53,54]. Nrf2 also regulates components of the thioredoxin system, including thioredoxin reductase, which maintains redox-sensitive proteins in a functional reduced state [55]. In the heart, mitochondrial thioredoxin reductase 2 limits excessive mitochondrial hydrogen peroxide emission and helps maintain mitochondrial redox control [56].

NAD(P)H oxidoreductase 1 (NQO1) reduces quinones through a two-electron reaction, thereby limiting redox cycling and the formation of additional reactive intermediates [57]. Glutathione, thioredoxin, and NQO1 therefore contribute not only to oxidant removal but also to protein redox regulation, detoxification, and preservation of cellular signaling.

Heme oxygenase-1 (HO-1) degrades heme into biliverdin, carbon monoxide, and ferrous iron, with biliverdin subsequently converted to bilirubin [58]. Carbon monoxide can support vasoregulatory and anti-inflammatory signaling, while biliverdin and bilirubin provide antioxidant protection. The released iron must be safely sequestered because free iron can promote further oxidative injury [58]. HO-1 induction is therefore potentially protective, but its biological effect depends on coordinated handling of all products generated during heme degradation.

Nrf2 also influences metabolic pathways that generate NADPH and reducing equivalents required by the glutathione and thioredoxin systems [59]. Nrf2 activation consequently changes the cellular ability to produce, use, and regenerate antioxidant capacity rather than simply increasing the removal of existing ROS.

#### 3.1.3. Crosstalk Between Nrf2 and Inflammatory Signaling

The Nrf2 and NF-κB pathways provide an important point of communication between cellular redox adaptation and inflammation. They should not be regarded as simple molecular opposites because both pathways can be activated by the same stressor, and the direction of their interaction varies with cell type, stimulus, and timing [20,21].

Nrf2 can attenuate NF-κB signaling indirectly by increasing glutathione-dependent defenses, HO-1 expression, and other systems that reduce the redox signals sustaining inflammatory activation [21,60]. More direct regulatory interactions have also been described. Keap1 can promote the ubiquitin-dependent degradation of IκB kinase β (IKKβ), thereby limiting activation of the NF-κB pathway [21,60]. Nrf2 and the NF-κB p65 subunit can also compete for the transcriptional coactivators CBP/p300, while p65 can recruit histone deacetylase 3 to ARE-containing regulatory regions and suppress Nrf2-dependent transcription [21,60]. Inflammatory signaling can therefore modify the Nrf2 response, while Nrf2-dependent adaptation can alter both the redox and transcriptional conditions supporting NF-κB activity.

Functional interaction between these pathways has also been observed in compound-specific experimental models. Salvianolic acid A produced coordinated changes in Akt, Nrf2, and NF-κB signaling in a cardiomyocyte lipotoxicity model [63]. Docosahexaenoic acid reduced inflammatory signaling partly through interaction between the Nrf2/HO-1 and IKK/NF-κB pathways in macrophages [64]. Such findings demonstrate functional coupling in particular experimental settings but do not establish that the same molecular interaction operates in every cardiovascular cell or disease.

A reduction in NF-κB activity following Nrf2 activation should therefore not automatically be interpreted as direct molecular inhibition. Lower NF-κB activity may result from reduced ROS generation, altered mitochondrial function, improved cellular metabolism, or regulation at the Keap1–IKKβ and CBP/p300 levels [21,60]. Direct mechanistic claims require pathway-specific perturbation rather than simultaneous measurement of Nrf2- and NF-κB-related markers. The distinction is particularly important when interpreting the broad pathway effects attributed to phytochemicals [15].

#### 3.1.4. Phytochemical Modulation of Nrf2

Phytochemical studies provide different levels of evidence for Nrf2 involvement. Increased nuclear accumulation of Nrf2 or expression of Nrf2-responsive genes indicates pathway activation. Stronger evidence is obtained when genetic or pharmacological disruption of Nrf2 weakens both target-gene induction and the associated cardiovascular protection [21,61,65].

Sulforaphane provides a well-characterized example of direct electrophilic target engagement [61,66,67]. Evidence for the functional importance of this mechanism extends beyond pathway activation. In a mouse model of angiotensin II-induced cardiomyopathy, sulforaphane activated Nrf2 and reduced cardiac oxidative injury and remodeling, whereas its protective effects were markedly diminished in Nrf2-deficient mice [62]. A separate study similarly showed that sulforaphane reduced angiotensin II-induced aortic inflammation and injury in wild-type mice but failed to provide comparable protection or induce Nrf2 target genes in Nrf2-deficient animals [68]. These loss-of-function findings support a necessary role for Nrf2 in the protection observed in these models.

Resveratrol also provides evidence of Nrf2-dependent vascular protection. In human coronary arterial endothelial cells, resveratrol increased Nrf2 transcriptional activity and the expression of NQO1, GCLC, and HO-1 [69]. Nrf2 knockdown or Keap1 overexpression attenuated target-gene induction and reduced the protective effect of resveratrol against oxidative stress [69]. Resveratrol-mediated improvement of vascular oxidative stress, vasodilation, and endothelial cell survival was also diminished in Nrf2-deficient mice [69].

Target-specific evidence has also been reported for quercetin. In oxidized LDL-treated macrophages, quercetin promoted the dissociation of Nrf2 from Keap1, increased Nrf2 nuclear translocation, and reduced ROS accumulation, NLRP3 activation, and pyroptosis [70]. Pharmacological inhibition of Nrf2 weakened these effects. Mutation of Arg483 in Keap1 also reduced the cellular effects of quercetin and its anti-atherosclerotic activity in ApoE-deficient mice, providing evidence that modulation of the Keap1–Nrf2 interaction contributed to the observed protection [70].

These studies distinguish Nrf2 pathway activation from stronger evidence of Nrf2 dependence. Increased expression of HO-1, NQO1, or other Nrf2-responsive proteins alone demonstrates pathway association, whereas loss of protection after Nrf2 disruption provides more direct mechanistic support. Nrf2 dependence in a specific model does not establish that Nrf2 is the only target of a phytochemical. The broader multi-pathway actions of these compounds are examined in Section 4.

#### 3.1.5. Context-Dependent Effects and Limitations

The main cytoprotective role of Nrf2 in experimental cardiovascular disease does not mean that more Nrf2 activity is always better. Its effects depend on the strength and duration of activation, metabolic state, cell type, and disease context [51].

Cancer biology provides the clearest warning about persistent Nrf2 activation. Loss-of-function changes in KEAP1 can lead to sustained Nrf2 activity that helps malignant cells tolerate oxidative stress, adapt their metabolism, and resist cell death [60]. This is not equivalent to brief dietary or pharmacological activation in cardiovascular tissue, but it shows that a protective stress pathway can have different effects when it remains active outside normal control.

This caution also applies to long-term cardiovascular prevention. The aim should be a regulated stress response, not maximal or continuous Nrf2 activation. This view is consistent with redox hormesis, in which a moderate stress signal can increase cellular resilience while sustained activation may have context-dependent effects [16,51].

Human data on sustained Nrf2 activity in cardiovascular disease remain limited. A small cross-sectional study in men with abdominal aortic aneurysm found stage-related differences in circulating markers associated with the Nrf2 antioxidant system, but it could not establish Nrf2 activity in tissues or causality [71]. The study and its limitations regarding the AAA phenotype are discussed in Section 5.2.

Circulating NRF2-related measures, tissue Nrf2 activity, target-gene activation, and functional antioxidant capacity are different readouts and should not be treated as equivalent.

Overall, Nrf2 is a credible regulator of cardiovascular stress adaptation and a plausible phytochemical target [51]. However, pathway activation is not a surrogate for cardiovascular efficacy. Human studies must show that Nrf2 is engaged at an achievable exposure and that this change improves vascular or myocardial function. This integrated Nrf2–ARE–NF-κB relationship, including the hormesis caveat, is summarized in Figure 1.

### 3.2. NADPH Oxidases and Redox Amplification

As introduced in Section 2.3, NADPH oxidases occupy a distinctive position in cardiovascular redox biology because ROS generation is their primary enzymatic function [46]. Individual NOX isoforms differ in cellular distribution, subcellular localization, activation mechanisms, and predominant ROS products, allowing NOX-derived ROS to participate in both physiological signaling and cardiovascular injury [44,72,73]. Consequently, NOX enzymes cannot be treated as a single uniform source of harmful ROS. Therapeutic strategies should selectively limit pathological NOX activity while preserving localized redox signaling required for normal cardiovascular function [42,43,74].

#### 3.2.1. Cardiovascular NOX Isoforms

NOX1 and NOX2 predominantly generate superoxide and generally require interactions with regulatory proteins for full activation [46,73]. NOX2 was initially characterized in phagocytes, where rapid superoxide production supports antimicrobial defense [46]. In vascular cells, NOX1 and NOX2 respond to stimuli such as angiotensin II, inflammatory mediators, growth factors, and mechanical stress [45,75]. Sustained renin–angiotensin system-dependent NOX activation can promote endothelial dysfunction, vascular remodeling, and fibroblast activation [75,76].

NOX4 differs in both its regulation and predominant ROS product. NOX4 is not acutely gated by classical activation signals; instead, its output is determined mainly by its abundance and subcellular localization [77,78]. The predominant detectable product of NOX4 is hydrogen peroxide, which is more stable than superoxide and can participate in redox-sensitive signaling [77,78]. NOX4-derived hydrogen peroxide has been associated with adaptive signaling in some settings but with fibrosis, hypertrophy, and cellular injury in others [44,72,77]. Changes in NOX4 expression alone therefore cannot be classified as either protective or harmful without considering cell type, localization, disease stage, and the magnitude and duration of ROS production.

NOX5 is activated directly by calcium and links changes in intracellular calcium to ROS generation in vascular cells [79]. In porcine coronary smooth-muscle cells, NOX5-dependent signaling was required for increased expression of the calcium-activated potassium channel KCNN4 [80]. NOX5 is also expressed in human vascular smooth-muscle and endothelial cells [81]. Unlike NOX1, NOX2, and NOX4, however, NOX5 is absent from the genomes of conventional mice and rats. Mice engineered to express human endothelial NOX5 develop age-dependent endothelial dysfunction and hypertension associated with eNOS uncoupling [82]. Standard rodent models therefore cannot fully reproduce NOX5-dependent mechanisms relevant to human vascular disease.

Isoform-specific differences have direct implications for therapeutic interpretation. Suppression of NOX1- or NOX2-derived superoxide may have different consequences from inhibition of context-dependent NOX4 signaling. Findings obtained exclusively in conventional rodents also cannot establish activity against NOX5-dependent human vascular dysfunction. Identification of the relevant NOX isoform is therefore necessary before a reduction in NOX activity can be interpreted as a specific therapeutic mechanism [42,43,74].

#### 3.2.2. NOX–eNOS Interaction

NOX-derived superoxide can rapidly react with NO to form peroxynitrite, thereby disrupting endothelial NO signaling [33,38]. This reaction simultaneously decreases NO bioavailability and increases oxidative and nitrosative stress [33]. Peroxynitrite can oxidize tetrahydrobiopterin (BH_4_), an essential cofactor required for coupled eNOS activity [34,38]. Loss of BH_4_ impairs electron transfer within eNOS and promotes enzymatic uncoupling, leading eNOS to generate superoxide rather than NO [34].

NOX activation can therefore recruit eNOS as an additional ROS source rather than merely adding superoxide to the vascular environment. The resulting increase in superoxide further reduces NO availability and favors additional peroxynitrite formation, creating a self-amplifying redox disturbance [33,34]. Impaired NO signaling subsequently contributes to abnormal vascular tone, platelet activation, leukocyte adhesion, and vascular smooth-muscle growth [33,34]. The regulation and functional consequences of eNOS uncoupling are discussed further in Section 3.4. This NOX-driven eNOS uncoupling and its convergence with NLRP3 priming and activation are summarized in Figure 2.

#### 3.2.3. NOX–Mitochondrial Crosstalk

NOX enzymes and mitochondria participate in reciprocal redox communication rather than functioning as independent ROS sources. NOX-derived ROS can alter mitochondrial membrane potential, calcium handling, electron transport, and mitochondrial permeability, thereby increasing mitochondrial ROS production. Mitochondrial ROS can, in turn, activate redox-sensitive kinases and other signaling mechanisms that enhance NOX activity [83]. The resulting feedback can recruit additional ROS sources, including uncoupled eNOS, and sustain oxidative stress after the initiating stimulus has diminished [83].

This interaction changes oxidative stress from a source-specific disturbance into a self-reinforcing cellular state. An increase in total cellular ROS may originate from primary NOX activation, mitochondrial dysfunction, eNOS uncoupling, or communication among all three systems. Parallel reductions in NOX activity and mitochondrial ROS after an intervention therefore do not demonstrate direct inhibition of both sources. Suppression of one component may indirectly reduce the other by interrupting reciprocal activation [83]. Mechanistic studies should consequently determine the sequence of pathway changes before assigning independent activity against multiple ROS sources.

#### 3.2.4. Target-Specific Evidence for Phytochemical Modulation of NOX

Phytochemical studies provide different levels of evidence for NOX involvement. A reduction in total ROS provides only indirect evidence, as it does not identify the source of the responsible ROS. Changes in NOX expression provide evidence of pathway-associated regulation, whereas measurements of enzymatic activity, regulatory-subunit assembly, or responses to isoform-specific disruption provide greater mechanistic resolution [42,43,74].

Resveratrol is supported by evidence primarily based on changes in NOX expression. In human endothelial cells, resveratrol produced a concentration- and time-dependent reduction in NOX4 expression and an increase in SOD1 and GPx1 expression. These changes were accompanied by lower cellular oxidative stress, supporting coordinated regulation of ROS production and antioxidant defense [84]. The study did not demonstrate direct binding to or catalytic inhibition of NOX4. Its findings support transcriptional modulation of the NOX4-associated redox environment rather than direct inhibition of NOX4. Because NOX4 can have adaptive or harmful effects depending on the context, reduced NOX4 expression cannot be considered beneficial in isolation from the experimental setting [44,77,78].

Quercetin provides evidence at the levels of enzymatic activity and NOX regulatory machinery. Quercetin and its metabolites, isorhamnetin and kaempferol, inhibited membrane NADPH oxidase activity in vascular smooth-muscle cells from both normotensive and spontaneously hypertensive rats [85]. Their effects were greater than could be explained by direct superoxide scavenging, and kinetic analysis indicated noncompetitive inhibition of NADPH [85]. The inhibitory effects were similar in cells from normotensive and hypertensive animals rather than being restricted to the hypertensive condition.

In a human endothelial cell model, quercetin prevented the angiotensin II-induced increase in superoxide and reduction in NO availability [86]. Quercetin also prevented the increase in p47^phox^ mRNA and protein expression induced by angiotensin II [86]. The study demonstrated regulation of p47^phox^ expression rather than inhibition of its membrane translocation. Because p47^phox^ contributes to activation of NOX1- and NOX2-containing complexes, the findings identify involvement of NOX regulatory machinery but do not conclusively distinguish between the two catalytic isoforms [46,73].

Lower ROS levels after phytochemical treatment can arise through several mechanisms. A compound may alter NOX isoform expression, interfere with regulatory-subunit assembly, inhibit enzymatic activity, reduce an upstream stimulus, improve mitochondrial function, or increase ROS removal. Each mechanism can reduce oxidative stress but represents a different level of NOX selectivity [42,43,74]. Likewise, increased NO availability after treatment may result from reduced NOX-derived superoxide and preservation of eNOS coupling rather than direct stimulation of eNOS [33,34].

A mechanistically supported claim of NOX modulation should therefore identify the relevant isoform and cell type, distinguish enzyme abundance from activation, and measure the ROS produced. Evidence is strengthened when genetic or isoform-selective pharmacological perturbation links NOX modulation to reduced redox amplification and improvement in a relevant cardiovascular phenotype [42,43,74]. The broader pharmacology, metabolism, systemic exposure, and translational evidence for resveratrol, quercetin, and other phytochemicals are considered in Section 4.

### 3.3. The NF-κB–NLRP3–Pyroptosis Axis

The NLRP3 inflammasome connects cellular stress to innate immune activation in cardiovascular disease. It responds to signals associated with mitochondrial dysfunction, ionic disturbance, lysosomal injury, extracellular ATP, metabolic stress, and crystalline material [47,87,88]. Activation of the inflammasome leads to caspase-1-dependent maturation of IL-1β and IL-18 and gasdermin D-mediated pyroptosis [89,90]. NLRP3 therefore functions as an amplification system through which metabolic and oxidative injury is converted into a sustained inflammatory response.

#### 3.3.1. Redox-Sensitive NF-κB Signaling and NLRP3 Priming

NF-κB provides a major transcriptional link between inflammatory stimulation and NLRP3 activation. In resting cells, NF-κB dimers are retained in the cytoplasm by inhibitory IκB proteins. Toll-like receptor and cytokine-receptor signaling activates the IκB kinase complex, promotes IκB degradation, and permits NF-κB translocation to the nucleus [14,20]. Nuclear NF-κB increases the transcription of NLRP3 and pro-IL-1β, establishing the primed state required for efficient inflammasome activation [47,90].

Priming is not exclusively a change in gene expression. Post-translational modifications can also determine whether NLRP3 is competent to assemble. For example, BRCC3-mediated deubiquitination of NLRP3 promoted inflammasome activation and atherosclerosis in a model of TET2-mutant clonal hematopoiesis [91]. The primed state therefore reflects both increased availability of inflammasome components and molecular changes that permit subsequent assembly.

Redox signaling can influence NF-κB activation and NLRP3 priming, but ROS should not be interpreted as a universal activator of the assembled inflammasome. ROS inhibition reduced NLRP3 expression and impaired priming without preventing activation once an adequate priming program had been established [92]. Reduced NLRP3 expression after an intervention may therefore indicate suppression of NF-κB-dependent priming rather than direct inhibition of NLRP3 assembly.

#### 3.3.2. NLRP3 Assembly and Activation

After priming, diverse danger signals converge on cellular processes that permit NLRP3 assembly. Established activation-associated events include extracellular ATP/P2X7 signaling, potassium efflux, lysosomal disruption, mitochondrial dysfunction, and exposure to crystalline material [47,87,90]. Activated NLRP3 recruits the adaptor protein ASC, which forms oligomeric structures that provide a platform for pro-caspase-1 recruitment and activation [90].

Several of these processes are relevant to cardiovascular disease. Cholesterol crystals activate NLRP3 in macrophages and contribute to atherosclerotic lesion development [93]. Hypoxia-induced metabolic reprogramming can also promote NLRP3 activation in macrophages through PFKFB3-dependent glycolysis [94]. NLRP3 activation in cardiovascular tissues should therefore be interpreted in the context of the metabolic, lipid, and ionic environment in which inflammasome assembly occurs.

Mitochondrial injury provides another connection between redox disturbance and NLRP3 activation. Loss of mitochondrial integrity can alter membrane potential and electron transport while promoting mitochondrial ROS production and the release of mitochondrial components. Oxidized mitochondrial DNA released into the cytosol can bind to NLRP3 and facilitate inflammasome activation [95]. Mitochondrial ROS and damage-associated molecules can therefore create conditions that favor activation, although ROS alone does not constitute an obligatory second signal in every setting [92,95].

#### 3.3.3. Reactive Cysteines as Molecular Targets of Electrophilic Modulators of NLRP3

NLRP3 itself is increasingly recognized as an electrophile-sensitive protein, raising the possibility that some natural products may regulate inflammasome activity through covalent modification rather than solely through nonspecific ROS scavenging or suppression of upstream oxidative stress. Several reactive cysteine residues within NLRP3 can undergo electrophile-dependent modification, although the functional consequences and selectivity vary according to the compound and experimental system. The clearest natural-product example is oridonin, an electrophilic diterpenoid that covalently modifies Cys279 within the NACHT domain of NLRP3. This interaction disrupts NLRP3–NEK7 binding and consequently prevents inflammasome assembly rather than merely decreasing cellular ROS. Other electrophilic molecules have been reported to modify additional NLRP3 cysteines and interfere with ATPase activity or NEK7-dependent assembly, supporting the concept that NLRP3 functions, at least partly, as an electrophile-sensitive signaling hub. Importantly, however, this mechanism should not be extrapolated to phytochemicals such as curcumin, resveratrol, or quercetin simply because they reduce NLRP3 activation. For these compounds, much of the available evidence supports indirect regulation via NF-κB-dependent priming, mitochondrial ROS, P2X7 signaling, metabolic pathways, or Nrf2-dependent redox adaptation, rather than direct covalent modification of NLRP3. Thus, reduction of NLRP3 expression, caspase-1 activation, or IL-1β release should be distinguished from direct molecular engagement of NLRP3.

Importantly, the presence of redox-sensitive or electrophile-reactive cysteines in NLRP3 does not imply that cysteine modification is a universal mechanism of phytochemical inflammasome inhibition; evidence of covalent target engagement requires identification of the modified residue and demonstration that its modification alters NLRP3 assembly or function.

#### 3.3.4. Caspase-1 Activation, Cytokine Maturation, and Pyroptosis

Inflammasome assembly activates caspase-1, which processes pro-IL-1β and pro-IL-18 into their mature inflammatory forms [47,90]. Caspase-1 also cleaves gasdermin D, releasing its N-terminal pore-forming domain [96]. Gasdermin D fragments oligomerize in the plasma membrane, forming pores that facilitate cytokine release and disrupt ionic homeostasis. Extensive pore formation ultimately causes membrane failure and pyroptotic cell death [96].

Pyroptosis amplifies inflammation by combining cytokine release with the discharge of intracellular danger signals. In atherosclerotic plaques, macrophage pyroptosis can increase inflammatory mediators and cellular debris and contribute to expansion of the necrotic core [89,97,98]. Pyroptotic injury in endothelial cells, vascular smooth-muscle cells, and cardiomyocytes has also been implicated in vascular and myocardial pathology [89,99,100]. Elevated circulating gasdermin D has been reported in patients with acute myocardial infarction, although this association does not by itself identify the affected cell type or establish causality [101].

Measurement of NLRP3 expression alone therefore provides incomplete evidence of functional inflammasome inhibition. A more complete assessment should distinguish transcriptional priming from inflammasome assembly and evaluate ASC oligomerization, caspase-1 activation, IL-1β and IL-18 maturation, gasdermin D cleavage, and pyroptotic membrane injury [89,97,98,100].

These findings are mechanistically distinct from direct covalent inhibition of NLRP3. Although curcumin contains electrophilic α,β-unsaturated carbonyl groups capable of Michael-type reactions with protein thiols, the studies considered here do not establish NLRP3 cysteine modification as the mechanism responsible for its inflammasome effects. Curcumin should therefore be classified primarily as an upstream/pathway modulator in this context, unless direct engagement with NLRP3 is demonstrated.

#### 3.3.5. Cardiovascular and Clinical Relevance

Experimental evidence supports a causal role for NLRP3 signaling in atherosclerosis. Cholesterol crystals activated NLRP3 in macrophages, while deficiency of NLRP3-associated inflammasome components reduced atherosclerotic lesion development in mice [93]. NLRP3 activation can also promote a phenotypic change in vascular smooth-muscle cells that contributes to plaque progression [102].

Clinical trials establish inflammation as a modifiable component of residual cardiovascular risk. In CANTOS, canakinumab 150 mg reduced recurrent cardiovascular events but increased the incidence of fatal infection [103]. Low-dose colchicine reduced composite ischemic outcomes following myocardial infarction in COLCOT and in patients with chronic coronary disease in LoDoCo2 [104,105]. The overall priming-to-pyroptosis cascade, its phytochemical entry points, and this clinical validation are summarized in Figure 3.

These findings require target-specific interpretation. Canakinumab neutralizes IL-1β downstream of inflammasome activation, whereas colchicine affects several inflammatory processes. The trials support the clinical importance of cardiovascular inflammation but do not demonstrate that NLRP3 was the only relevant source of inflammation or that direct NLRP3 inhibition would produce identical benefits. The increased risk of fatal infection with canakinumab also illustrates the need to limit pathological inflammation without broadly impairing host defense [103].

#### 3.3.6. Target-Specific Evidence for Phytochemical Modulation of the NLRP3 Axis

Phytochemicals may act at several levels of the NF-κB–NLRP3–pyroptosis axis. They may reduce NF-κB-dependent priming, mitochondrial or metabolic stress, ATP/P2X7 signaling, inflammasome assembly, caspase-1 activation, gasdermin D cleavage, or cytokine release. Similar reductions in NLRP3 and IL-1β can therefore arise through pharmacologically different mechanisms.

Berberine provides evidence of upstream metabolic regulation. In db/db mice and high-glucose-treated H9C2 cells, berberine reduced mitochondrial ROS and decreased NLRP3, caspase-1, IL-1β, IL-18, and gasdermin D-associated pyroptosis [106]. Pharmacological modulation of mTOR altered the protective effects of berberine, supporting involvement of the mTOR–mitochondrial ROS axis [106]. The study links upstream metabolic regulation to reduced inflammasome activity and cardiac protection but does not demonstrate direct binding of berberine to NLRP3.

Curcumin has been reported to affect both priming- and activation-associated signaling. In PMA-differentiated macrophages, curcumin inhibited TLR4/MyD88/NF-κB signaling and reduced P2X7 receptor expression, NLRP3 expression, caspase-1 cleavage, and IL-1β release [107]. P2X7 knockdown also reduced inflammasome-associated responses, supporting involvement of this upstream pathway [107]. Because this experiment was conducted in a macrophage model rather than a cardiovascular disease model, it provides mechanistic support but does not independently establish cardiovascular protection.

Quercetin reduced atherosclerotic lesions and NLRP3-associated inflammation in ApoE-deficient mice [108]. In oxidized LDL-loaded macrophages, quercetin reduced galectin-3 expression, NLRP3 activation, and IL-1β secretion. Restoration of galectin-3 weakened these effects, supporting galectin-3 as an upstream component of quercetin-sensitive NLRP3 signaling [108]. These findings demonstrate pathway dependence more clearly than a reduction in NLRP3 expression alone, although they do not establish direct interaction between quercetin and NLRP3.

The selective NLRP3 inhibitor MCC950 provides a useful mechanistic comparator. MCC950 reduced IL-1β release and atherosclerotic lesion development in ApoE-deficient mice without suppressing the TNF-α response [109]. Phytochemical effects on upstream stress pathways should not be described as equivalent to selective NLRP3 inhibition unless direct target engagement or inhibition of inflammasome assembly is demonstrated.

Mechanistically strong evidence should identify the level at which an intervention acts and link that effect to downstream execution of the pathway. Appropriate assessment includes markers of priming, inflammasome assembly, caspase-1 activation, cytokine maturation, gasdermin D cleavage, pyroptotic injury, and a relevant cardiovascular phenotype. Compound-specific multi-target actions, exposure, and clinical evidence are considered separately in Section 4.

### 3.4. Endothelial Dysfunction as an Integrated Vascular Phenotype

The vascular endothelium is a principal site where oxidative and inflammatory signaling converge to drive clinically relevant cardiovascular dysfunction. Healthy endothelial cells regulate vascular tone, permeability, hemostasis, leukocyte trafficking, and vascular smooth-muscle behavior [110,111]. Nitric oxide (NO) is central to these functions because it promotes vasodilation while limiting platelet activation, leukocyte adhesion, and vascular smooth-muscle proliferation [110,111]. Endothelial dysfunction develops when NO signaling declines and the endothelial surface acquires a more inflammatory, adhesive, and prothrombotic phenotype [110].

Endothelial dysfunction represents more than a downstream marker of oxidative stress. It integrates changes in ROS production, eNOS activity, inflammatory signaling, and endothelial barrier function into a measurable vascular phenotype. This integrated role makes the endothelium particularly useful for assessing whether molecular effects attributed to phytochemicals produce meaningful changes in vascular physiology.

#### 3.4.1. Physiological Regulation of eNOS and NO Signaling

Endothelial nitric oxide synthase (eNOS) produces NO from L-arginine and molecular oxygen through a reaction that requires tetrahydrobiopterin (BH_4_) and coordinated electron transfer between the reductase and oxygenase domains of the enzyme [34,111]. Calcium–calmodulin binding, phosphorylation, subcellular localization, and interactions with regulatory proteins allow blood flow, shear stress, and receptor-mediated signals to regulate eNOS activity [34,111,112].

NO diffuses from endothelial cells into adjacent vascular smooth-muscle cells, where it activates soluble guanylyl cyclase and cyclic guanosine monophosphate-dependent signaling to promote relaxation [110,113]. Endothelial NO also restrains platelet activation, leukocyte adhesion, vascular smooth-muscle proliferation, and inflammatory signaling [110,111]. The protective endothelial phenotype depends on the production of biologically active NO rather than simply on the amount of eNOS protein present.

#### 3.4.2. eNOS Uncoupling and Redox Amplification

eNOS becomes uncoupled when electron transfer within the enzyme continues without efficient NO formation. Oxidation or depletion of BH_4_, limited availability of L-arginine, accumulation of endogenous eNOS inhibitors, and oxidative modification of the enzyme can promote this transition [34,38,112]. Uncoupled eNOS produces superoxide instead of NO, simultaneously reducing vasoprotective signaling and creating an additional source of ROS [33,34].

Superoxide generated by NOX enzymes, mitochondria, or uncoupled eNOS rapidly reacts with NO to form peroxynitrite [33,38,114]. Peroxynitrite formation consumes NO and promotes oxidation of proteins, lipids, and redox-sensitive cofactors, including BH_4_ [34,38,114]. BH_4_ oxidation further destabilizes eNOS coupling, producing a feed-forward cycle in which declining NO availability and increasing superoxide production reinforce one another [33,34,38].

Human vascular findings support this mechanism. In coronary arterioles obtained from patients with diabetes, increased peroxynitrite formation was associated with disruption of endothelial caveolae and caveolin-1 signaling, eNOS uncoupling, and impaired NO-mediated vasodilation [115]. Restoration of BH_4_ availability improved coronary arteriolar dilation in this experimental setting, linking the molecular disturbance to a functional vascular response [115].

Increased eNOS expression alone cannot be interpreted as evidence of restored endothelial NO signaling. Evaluation is strengthened by examining the BH_4_/BH_2_ balance, eNOS coupling, NO production, superoxide or peroxynitrite formation, and an endothelial functional outcome [34,116]. This distinction is particularly important when interpreting interventions that increase eNOS expression while leaving the surrounding oxidative environment unchanged.

#### 3.4.3. Endothelial Inflammatory Activation

Loss of NO signaling is accompanied by a change in the inflammatory behavior of endothelial cells. Cytokines, oxidized lipids, disturbed shear stress, and excessive ROS can activate redox-sensitive transcriptional pathways, including NF-κB, within the endothelium [22]. NF-κB-dependent signaling increases the expression of vascular cell adhesion molecule-1 (VCAM-1), intercellular adhesion molecule-1 (ICAM-1), E-selectin, and chemokines that recruit circulating leukocytes [22].

Selectins support the initial rolling of leukocytes along the endothelial surface, whereas interactions involving ICAM-1 and VCAM-1 promote firm adhesion and subsequent transendothelial migration [22]. Expression of these molecules is therefore not merely a circulating marker of inflammation. It represents an active change in endothelial behavior that controls the entry of inflammatory cells into the vascular wall.

Declining NO bioavailability further favors this transition because physiological NO normally limits leukocyte and platelet adhesion [110,111]. Recruited leukocytes then release additional ROS, cytokines, proteases, and other inflammatory mediators that can disrupt endothelial junctions and increase vascular permeability [22]. Oxidative stress and endothelial inflammation consequently reinforce one another without requiring either process to remain strictly upstream of the other.

#### 3.4.4. Integrated Functional Manifestations

The combined loss of NO-dependent vasodilation, increased endothelial adhesiveness, altered permeability, and enhanced platelet–vascular interactions results in the functional phenotype known as endothelial dysfunction [110]. This phenotype reflects the net vascular effect of several interacting mechanisms.

Flow-mediated dilation (FMD) is commonly used to assess this phenotype in humans. It measures conduit-artery dilation in response to increases in blood flow and shear stress and, under standardized conditions, exhibits a substantial NO-dependent component [117]. FMD is not, however, an exclusive measure of NO production, as procedural conditions and additional endothelial mediators can influence the response [117]. Impaired FMD is associated with cardiovascular risk, but it remains an intermediate vascular phenotype rather than a direct measure of cardiovascular events [110].

Endothelial function can therefore connect pathway-level observations with vascular physiology. Changes in Nrf2, NOX activity, inflammatory mediators, or oxidative biomarkers indicate biological activity, whereas improvement in NO-dependent vascular function demonstrates that these molecular changes are accompanied by a functional response. The two levels of evidence should remain distinct.

#### 3.4.5. Target-Specific Evidence for Phytochemical Modulation of Endothelial Function

The purpose of evaluating phytochemicals in this section is not to provide a compound-by-compound clinical review. The central question is whether modulation of redox and inflammatory targets is accompanied by restoration of endothelial NO signaling or vascular function.

Experimental studies of resveratrol have reported increased eNOS activity and NO bioavailability together with reduced endothelial oxidative stress and modulation of NOX-dependent signaling [118,119]. These observations support endothelial signaling as one component of resveratrol activity, although they do not establish eNOS or NOX as its only molecular targets. A meta-analysis of 17 randomized studies found that resveratrol supplementation was associated with increased FMD and reduced circulating ICAM-1, whereas VCAM-1 did not change significantly [120]. The human findings support a vascular functional signal but do not establish which molecular pathway produced that response.

Quercetin has similarly been associated with reduced vascular oxidant production, preservation of NO bioavailability, and improved endothelial responses in experimental models [121,122]. Because quercetin can influence NOX activity, antioxidant defenses, inflammatory signaling, and cellular metabolism, simultaneous changes in these pathways should not be interpreted as evidence that it acts directly on every measured target.

Clinical studies of curcumin preparations provide another phenotype-level example. A meta-analysis of five randomized trials reported improvement in brachial artery FMD after curcumin supplementation [123]. Improvement in FMD supports an effect on endothelial function but does not by itself demonstrate direct activation of eNOS, prevention of eNOS uncoupling, or inhibition of a specific ROS source.

Mechanistic attribution is stronger when phytochemical treatment improves NO production or endothelial vasodilation and the response is weakened by disruption of the proposed pathway. Human evidence is strengthened when clinically achievable exposure, pathway-related biomarkers, and standardized endothelial function are assessed within the same study. Detailed differences in phytochemical formulation, metabolism, bioavailability, and clinical outcomes are considered separately in the compound- and translation-focused sections of this review.

### 3.5. Integration of the Core Mechanistic Network

The pathways examined in Section 3.1, Section 3.2, Section 3.3 and Section 3.4 can be viewed at three connected levels. Nrf2, NOX enzymes, and mitochondria regulate the balance between adaptive redox signaling and oxidative stress [14,39,42]. NF-κB, NLRP3, and pyroptosis translate sustained redox and metabolic disturbances into inflammatory responses and cell injury [7,47]. The eNOS/NO system and the vascular endothelium integrate these molecular changes into functional outcomes, including altered vasodilation, leukocyte adhesion, and thrombogenicity [33,34,110].

This connectivity provides a basis for interpreting the broad molecular effects reported after phytochemical treatment [14,15,124,125]. A change in several pathway markers may reflect a limited number of initiating effects followed by propagation through the network. Evidence should therefore be distinguished as proximal target engagement, pathway dependence, downstream network response, or improvement in an integrated cardiovascular phenotype. Direct target engagement requires biochemical or cellular evidence that a compound interacts with the proposed target, whereas pathway dependence requires showing that selective pharmacological or genetic disruption substantially weakens the response [126]. When these conditions are not met, simultaneous changes across multiple markers are more accurately described as pathway-associated or coordinated responses rather than as direct multi-target activity.

Section 3 establishes the mechanistic framework for interpreting phytochemical effects throughout the remainder of the review. The integrated network is summarized in Figure 4. The compound-centered sections that follow examine whether these pathway-associated effects occur at exposures achievable in humans and whether they are accompanied by target engagement and meaningful cardiovascular responses [15,127,128,129]. Separating these levels of evidence prevents broad preclinical pathway changes from being interpreted prematurely as clinically relevant cardioprotection.

## 4. Major Phytochemical Classes and Their Cardiovascular Evidence

This section is organized by phytochemical class but does not aim to provide an exhaustive catalog of all plant-derived compounds associated with cardiovascular health. Priority is given to phytochemicals with evidence of modulating the core redox–inflammatory pathways defined in Section 3, including Nrf2, NOX enzymes, NF-κB–NLRP3 signaling, mitochondrial redox regulation, and eNOS/NO homeostasis [14,15]. Compounds supported mainly by broader cardiovascular, metabolic, or endothelial findings are included as complementary evidence and are discussed more briefly.

For the pathway-focused compounds, the discussion considers the proposed initiating mechanism, its position within the redox–inflammatory network, evidence of pathway dependence, achievable human exposure, and cardiovascular relevance. For the supporting compounds, greater emphasis is placed on the observed cardiovascular phenotype and on the limitations of connecting that phenotype to a specific molecular target. This hierarchy prevents general antioxidant or cardiometabolic associations from being treated as equivalent to pathway-specific mechanistic evidence [15,127,128,130].

For mechanistic interpretation, we distinguish three fundamentally different modes of phytochemical action. First, electrophilic compounds may directly modify nucleophilic residues in target proteins, particularly reactive cysteines, as exemplified by sulforaphane–Keap1 and, among natural NLRP3 inhibitors, oridonin–NLRP3 interactions. Second, phytochemicals may regulate target abundance, localization, enzymatic activity, protein–protein interactions or upstream signaling without demonstrated covalent target engagement. Third, compounds may chemically react with ROS or other reactive species; however, direct scavenging observed in cell-free systems should not automatically be considered the dominant mechanism in vivo because achievable concentrations, metabolism, compartmentalization, and endogenous antioxidant systems substantially constrain stoichiometric radical scavenging. Throughout this section, these levels of evidence are distinguished to avoid equating downstream reductions in ROS or inflammatory markers with direct molecular target engagement.

### 4.1. Flavonoids

Flavonoids are chemically diverse polyphenols that differ in absorption, metabolism, molecular activity, and cardiovascular evidence [15,127,128]. Quercetin is emphasized as a pathway-focused example because experimental studies connect it to NOX-derived ROS, Nrf2, inflammatory signaling, and eNOS/NO homeostasis. EGCG has extensive mechanistic and preclinical evidence involving redox-sensitive and endothelial pathways, but equivalent target engagement has been less clearly demonstrated in humans. Anthocyanins and other flavan-3-ols are discussed primarily as supporting evidence, as their strongest human findings involve vascular function and cardiometabolic markers rather than direct confirmation of the core molecular pathways.

#### 4.1.1. Quercetin and Related Flavonols

Quercetin is a flavonol present in onions, apples, berries, tea, and numerous other plant foods [131,132]. Experimental cardiovascular studies associate quercetin with reduced NOX-related oxidative stress, preservation of eNOS/NO signaling, activation of Nrf2-responsive defense, and attenuation of inflammatory pathways [121,122,133,134,135]. These reported actions position quercetin at several points in the redox–inflammatory network defined in Section 3.

Quercetin has been experimentally associated with reduced vascular oxidative stress and improved endothelial function, although a direct dependence on a specific NOX isoform has not been consistently established across models [121,122,134,135].

Quercetin has also been linked to Nrf2 activation and reduced NF-κB-dependent inflammation [121,133,134]. These responses may reflect coordinated improvement in cellular redox control rather than independent direct actions on both pathways. Increased endogenous defense can create a less oxidizing intracellular environment, which may subsequently reduce activation of redox-sensitive inflammatory signaling. Studies measuring Nrf2 and NF-κB markers at a single time point cannot determine which response occurred first.

Oxidized LDL-induced endothelial and macrophage senescence provides a more disease-specific example. Quercetin reduced cellular senescence in association with p16/p21, p53/SERPINE1, and AMPK/mTOR signaling in an experimental model [136]. This finding connects metabolic and stress-response pathways to endothelial and macrophage dysfunction relevant to atherosclerosis. Its broader importance will depend on replication, pathway disruption, and confirmation at exposures achievable in vivo.

Human evidence is more limited than the experimental mechanistic literature. A meta-analysis of 17 randomized trials found modest reductions in systolic and diastolic blood pressure after quercetin supplementation, while overall lipid and glucose concentrations did not change significantly [137]. Another meta-analysis involving participants with metabolic syndrome and related disorders reported a small reduction in systolic blood pressure but no significant effects on diastolic blood pressure, VCAM-1, or ICAM-1 [138]. These findings support a possible vascular effect but do not confirm that NOX, Nrf2, NF-κB, or eNOS was responsible for the response.

Bioavailability further limits direct translation. Quercetin occurs in foods as multiple glycosides and is extensively transformed during intestinal absorption and phase II metabolism [128,132]. Circulating glucuronidated, sulfated, and methylated metabolites differ chemically from the unconjugated quercetin commonly used in cell experiments [132]. Interindividual variation in absorption, metabolism, and gut microbial transformation results in substantial differences in systemic exposure following the same oral dose [127,132].

Quercetin is retained as a core phytochemical in this review because its proposed actions intersect with several selected pathways. Its mechanistic relevance is stronger than its clinical validation. Future studies need to determine whether the circulating human metabolites reproduce the NOX, Nrf2, inflammatory, and endothelial effects attributed to unconjugated quercetin.

#### 4.1.2. EGCG and Other Tea Catechins

Epigallocatechin-3-gallate (EGCG) is a major catechin in green tea and has been widely investigated in cardiovascular and metabolic models [139,140,141]. Experimental studies associate EGCG with endothelial NO production, mitochondrial regulation, Nrf2-responsive defense, and attenuation of inflammatory signaling [139,142,143]. The relative importance of these pathways varies by model, concentration, and timing.

Endothelial mechanisms are particularly relevant. EGCG-induced vascular responses have been linked to eNOS activation and NO-dependent vasodilation in experimental systems [135,142,143]. Reduced oxidative consumption of NO may also contribute to improved endothelial function. Evidence that EGCG affects both ROS-related and eNOS-related markers does not establish separate direct actions; preservation of NO may follow from a primary reduction in vascular redox stress.

Preclinical cardiac evidence includes models of myocardial ischemia–reperfusion injury and infarction. A meta-analysis of animal studies reported cardioprotective effects of EGCG in these settings [144]. Proposed mechanisms include reduced oxidative injury, inflammatory activation, mitochondrial dysfunction, and cardiomyocyte death [142,144]. Variation in animal species, dosing, route of administration, and ischemia protocols limits the estimation of effective human exposure [144].

Human vascular evidence demonstrates the importance of distinguishing an isolated compound from its food source. In a randomized crossover study, green tea improved flow-mediated dilation, whereas green tea extract and isolated EGCG containing the same nominal EGCG dose did not [145]. Plasma EGCG concentration was highest after isolated EGCG, despite the absence of an FMD response [145]. Other tea components, metabolites, or interactions within the beverage may have contributed to the vascular effect.

The green-tea study cautions against assigning a food-level cardiovascular response to its most prominent phytochemical. Experimental EGCG activity remains relevant to the selected redox and endothelial pathways, but evidence from tea cannot automatically validate an isolated-EGCG mechanism. Human studies need to measure catechin metabolites, pathway-matched biomarkers, and vascular function within the same intervention.

#### 4.1.3. Anthocyanins and Flavan-3-ols

Anthocyanins are pigmented flavonoids found in berries, grapes, cherries, and other red, blue, or purple plant foods. Experimental studies associate them with redox-sensitive signaling, endothelial protection, and reduced vascular-cell senescence [125,146]. Direct pathway-dependence experiments involving Nrf2, NOX, NF-κB–NLRP3, or eNOS remain less consistent than the broader evidence for vascular effects.

Systematic reviews of randomized trials have evaluated anthocyanin-rich foods and extracts in relation to flow-mediated dilation, blood pressure, lipid profiles, and other cardiometabolic markers [147,148,149,150]. The most consistent signals involve selected measures of endothelial or vascular function, although effect sizes and statistical consistency vary across interventions [147,149,150]. Prospective cohort studies have also reported associations between higher anthocyanin intake and lower cardiovascular risk, but observational findings cannot establish a causal effect of anthocyanins [151].

Flavan-3-ols occur in cocoa, tea, apples, grapes, and related foods. A meta-analysis of randomized trials reported modest improvements in blood pressure and endothelial function, with responses varying according to dose, baseline risk, and intervention matrix [152]. Cocoa products differ in flavanol preservation, processing, sugar, fat, and total energy content [153]. Clinical findings from cocoa or chocolate cannot be generalized to purified flavan-3-ols without accounting for these differences.

Anthocyanins also undergo extensive degradation, phase II conjugation, and microbial transformation [154,155]. After berry consumption, intact anthocyanin concentrations are generally low, while several colonic and phenolic metabolites may contribute to systemic exposure [154,155]. Experimental studies using high concentrations of intact anthocyanins may not reproduce the molecular environment present in human circulation.

Chocolate and wine provide additional examples of food-matrix complexity. Chocolate contains variable amounts of cocoa flavanols together with sugar and fat, while wine combines polyphenols with ethanol [153,156]. A narrative review described potentially favorable cardiovascular associations for both foods but concluded that the evidence remained controversial and was not confirmed by a formal GRADE assessment [156]. Wine-related outcomes cannot be used as evidence for resveratrol in isolation, and chocolate outcomes cannot be attributed solely to a single cocoa flavanol.

Anthocyanins and flavan-3-ols strengthen the human vascular component of this review. Their evidence supports the cardiovascular relevance of endothelial function, vascular tone, and dietary polyphenol metabolism. They are treated as complementary evidence because the connection between the observed human phenotype and a defined initiating node in the core redox–inflammatory network remains less certain.

### 4.2. Stilbenes and Phenolic Acids

Resveratrol receives primary attention in this subsection because it has been linked to several components of the core redox–inflammatory network. Phenolic acids and olive-derived phenolics are included as supporting examples because their cardiovascular evidence is more often based on dietary exposure, endothelial outcomes, and circulating metabolites than on pathway-specific causal studies.

#### 4.2.1. Resveratrol

Resveratrol is a stilbene found in grapes, berries, peanuts, wine, and related dietary sources [157,158]. Experimental studies have associated resveratrol with eNOS/NO signaling, mitochondrial function, SIRT1- and AMPK-related metabolism, oxidative stress, and inflammatory regulation [118,119,157,158,159]. Increased eNOS activity and preservation of NO bioavailability connect resveratrol to the endothelial mechanisms described in Section 3.4 [118,119]. Changes in mitochondrial metabolism and SIRT1/AMPK signaling may also reduce the metabolic and oxidative stimuli that sustain NF-κB and inflammasome activity [158,159].

Reported reductions in NF-κB signaling, inflammatory mediators, and oxidative injury place resveratrol within the redox–inflammatory network, but they do not identify a single universal initiating target [157,158,159]. The sequence may differ according to cell type and disease context. Improved mitochondrial function may reduce ROS-dependent inflammatory signaling, whereas preservation of endothelial NO may, in turn, limit leukocyte adhesion and vascular inflammation.

Cardiac fibrosis illustrates how this pathway effects may converge on a cardiovascular phenotype. In cellular and animal models, resveratrol has reduced fibroblast activation, extracellular-matrix deposition, oxidative injury, and inflammatory signaling [160,161,162]. Proposed mechanisms include SIRT1/AMPK-related metabolic regulation and attenuation of TGF-β/Smad-dependent profibrotic responses [160]. The evidence supports a plausible link between redox–inflammatory modulation and cardiac remodeling, although most mechanistic studies remain preclinical [160,162].

Human studies have reported variable effects on endothelial function, inflammatory markers, lipid and glucose regulation, and clinical status in coronary artery disease and heart failure [161,162,163,164,165]. A meta-analysis in coronary artery disease found reductions in selected inflammatory markers, but the included studies differed in dose, duration, patient characteristics, and background treatment [165]. Consistent evidence of reduced cardiac fibrosis, heart failure progression, or major cardiovascular events has not yet been established [161,162,163,164,165].

Pharmacokinetics remain central to the interpretation of these mechanisms. Oral resveratrol undergoes rapid glucuronidation and sulfation, resulting in low circulating concentrations of the unchanged parent compound [166]. Many cellular studies use parent resveratrol concentrations that are difficult to reproduce following oral administration [163,166]. Biological activity in humans may involve conjugated metabolites, local tissue metabolism, or transient gastrointestinal and hepatic exposure [127,166]. Claims involving SIRT1, mitochondrial signaling, NF-κB, or eNOS are strongest when the administered formulation, circulating molecular species, pathway response, and cardiovascular phenotype are evaluated together.

Unlike sulforaphane-mediated Keap1 modification, direct covalent engagement of a specific redox–inflammatory target has not been established as the dominant cardiovascular mechanism of resveratrol; its effects are better supported as pathway-level regulation involving Nrf2-dependent transcription, NOX expression/activity, mitochondrial signaling, and endothelial NO homeostasis rather than simple stoichiometric ROS scavenging.

#### 4.2.2. Phenolic Acids and Olive-Derived Phenolics

Phenolic acids occur in fruits, whole grains, honey, coffee, and other plant foods [167,168,169,170]. Their absorption, conjugation, and microbial transformation produce several circulating molecular species [127,167,168,169,170,171,172]. Food interventions may consequently affect cardiovascular biology through parent compounds, phase II conjugates, microbiota-derived metabolites, or other constituents of the food matrix.

Hydroxytyrosol and related olive phenolics have been associated with changes in LDL oxidation, platelet activity, inflammatory markers, and endothelial function [171,172,173,174,175]. Hydroxytyrosol is absorbed but undergoes extensive metabolism after oral administration [171]. Tyrosol can also be converted endogenously into hydroxytyrosol, adding another source of interindividual variation in exposure [174]. Multi-omic analysis has identified heterogeneous platelet and molecular responses following hydroxytyrosol administration [173].

These findings complement the pathway-focused discussion by showing that changes in oxidative or vascular biomarkers can occur after dietary phenolic exposure. Direct dependence on Nrf2, NOX, NF-κB–NLRP3, or eNOS has been demonstrated less consistently than for the primary compounds reviewed in this section. Benefits associated with extra virgin olive oil also cannot be assigned exclusively to hydroxytyrosol because the intervention contains fatty acids and multiple minor bioactive components [175].

### 4.3. Curcuminoids

Curcuminoids are dominated in the cardiovascular literature by a single well-characterized compound, curcumin, discussed below.

Curcumin is included as a principal mechanistic example because experimental studies repeatedly connect it to Nrf2, NF-κB, NLRP3, mitochondrial stress, and endothelial signaling [107,176,177,178,179]. Its reported effects converge on the same redox–inflammatory network described in Section 3, although the initiating molecular mechanism remains less clearly defined than the Keap1 modification produced by sulforaphane.

Curcumin has increased Nrf2-responsive defense and reduced NF-κB-dependent inflammatory signaling in cardiovascular and metabolic models [177,178,179]. As discussed in Section 3.3.5, curcumin also inhibited NLRP3 activation in macrophages through signaling involving TLR4/MyD88/NF-κB and P2X7R [107]. Preclinical studies of diabetic cardiomyopathy additionally report reductions in oxidative stress, inflammation, fibrosis, and cardiac dysfunction [176]. Together, these effects suggest that curcumin may interrupt communication among metabolic stress, inflammatory priming, inflammasome activity, and tissue remodeling.

Changes in several pathways after treatment do not establish direct binding of curcumin to every altered target. Activation of an upstream adaptive response or reduction of mitochondrial stress could produce secondary changes in NF-κB, NLRP3, and endothelial signaling. Temporal analysis, selective inhibition, and genetic disruption are needed to identify the initiating event. Evidence of Nrf2 activation or NLRP3 suppression alone demonstrates pathway association, not pathway dependence.

Translation is also constrained by exposure. Native curcumin has low aqueous solubility, limited absorption, rapid metabolism, and extensive conjugation [180]. Turmeric powders, standardized extracts, piperine combinations, phospholipid complexes, micelles, and nanoparticle formulations generate different pharmacokinetic profiles [180,181,182]. Each formulation represents a distinct intervention and may not engage the same pathways at the same magnitude.

Clinical studies have reported changes in selected cardiometabolic and vascular markers. In a small trial involving overweight and obese adolescent girls, favorable within-group changes were observed in several anthropometric and lipid measurements, but adjusted comparisons between curcumin and placebo were not significant [183]. As introduced in Section 3.4.5, meta-analyses have reported improvement in flow-mediated dilation after curcumin supplementation; additional analyses indicate that pulse-wave velocity, augmentation index, endothelin-1, and soluble adhesion molecules show less consistent responses [123]. These results provide preliminary support for an endothelial phenotype but do not confirm the Nrf2, NF-κB, or NLRP3 mechanisms identified experimentally.

A pathway-focused curcumin study would need to establish systemic exposure to the relevant curcuminoid species, demonstrate engagement of a prespecified redox–inflammatory pathway, and connect that response to vascular or myocardial function. Without these elements, broad changes in oxidative or inflammatory biomarkers remain compatible with several possible mechanisms.

### 4.4. Carotenoids

Carotenoids are included mainly because lycopene and astaxanthin have been associated with cardiovascular risk markers and oxidative phenotypes. Direct causal evidence involving the specific Nrf2, NOX, NF-κB–NLRP3, or eNOS pathways emphasized in this review is less developed than for quercetin, curcumin, sulforaphane, or resveratrol.

Carotenoids are lipid-soluble pigments whose absorption depends on food processing, dietary fat, micelle formation, intestinal transport, and baseline carotenoid status [184,185,186]. Co-ingested fat, particularly unsaturated fat, increases carotenoid bioaccessibility and absorption [186]. Circulating carotenoid concentration is consequently more informative than nominal intake when evaluating a potential biological effect.

Lycopene has been studied in relation to lipid oxidation, inflammation, blood pressure, and endothelial function [184,185,187,188]. Meta-analyses of tomato products and lycopene supplements report favorable changes in some cardiovascular risk markers, but results differ by intervention and endpoint [185,187,188]. Tomato foods contain additional carotenoids, vitamins, minerals, and phenolic compounds, making it difficult to assign the entire response to lycopene [184,187,188]. Observational associations between higher lycopene exposure and lower cardiovascular risk cannot establish causality [189,190].

Astaxanthin has been linked experimentally to membrane protection, mitochondrial stress regulation, and inflammatory signaling [191,192]. Human studies remain small and heterogeneous. Trials have evaluated cardiometabolic markers in firefighters and in men with obesity receiving both astaxanthin and high-intensity training [193,194]. The combined exercise intervention prevents clear attribution of the response to astaxanthin [194].

Carotenoid findings support the broader relevance of lipid oxidation, mitochondrial stress, and vascular function to cardiovascular pathology. They currently provide limited evidence for direct modulation of the core mechanistic pathways selected for this review. Broader analyses of lipid-soluble antioxidant supplementation also indicate that responses differ by compound, dose, population, and outcome [195].

### 4.5. Organosulfur Compounds

Organosulfur compounds in the cardiovascular redox–inflammatory literature are represented almost entirely by a single well-characterized isothiocyanate, sulforaphane, discussed below.

Sulforaphane is one of the clearest examples of a phytochemical engaging with a core pathway reviewed in Section 3. It is an electrophilic isothiocyanate formed from glucoraphanin, a glucosinolate abundant in broccoli sprouts and other cruciferous vegetables [196,197,198]. Sulforaphane modifies redox-sensitive Keap1 cysteine residues, reduces Keap1-mediated Nrf2 degradation, and increases transcription of Nrf2-responsive cytoprotective genes [66,67,197,198,199].

Nrf2 activation can strengthen glutathione, thioredoxin, NQO1, HO-1, and related cellular defense systems. The resulting improvement in redox buffering may influence mitochondrial function, endothelial NO signaling, and inflammatory activation at several downstream points [66,67,199,200]. Experimental cardiometabolic studies have associated sulforaphane with improved endothelial protection, metabolic regulation, and attenuation of inflammatory signaling [199,200]. Studies in which Nrf2 disruption weakens protection provide stronger mechanistic support than the measurement of Nrf2-responsive proteins alone.

Exposure depends on precursor conversion. Plant myrosinase converts glucoraphanin into sulforaphane, and its activity is altered by heating, storage, processing, and formulation [196,198,201]. Intestinal microorganisms can provide partial conversion when plant myrosinase is inactive, but exposure becomes more variable [196,198]. A human crossover study found substantially greater urinary recovery of sulforaphane metabolites after a sulforaphane-rich broccoli sprout beverage than after a glucoraphanin-rich beverage [202]. Preservation of residual myrosinase during processing also increases and accelerates systemic isothiocyanate exposure [201].

Broccoli foods, glucoraphanin supplements, myrosinase-containing preparations, and stabilized sulforaphane products cannot be treated as equivalent interventions. Measurement of plasma or urinary sulforaphane metabolites confirms internal exposure. Induction of NQO1, HO-1, or another Nrf2-responsive product provides a separate measure of target engagement. Vascular or myocardial endpoints are then needed to determine whether pathway activation produces a relevant cardiovascular effect.

The direct Keap1–Nrf2 relationship gives sulforaphane high mechanistic relevance to this review, but its human cardiovascular evidence remains limited compared with its preclinical literature [67,199]. Clinical research commonly examines metabolic, inflammatory, or antioxidant biomarkers rather than cardiovascular events. Sulforaphane illustrates a well-defined initiating mechanism whose clinical cardiovascular significance still requires stronger confirmation.

### 4.6. Alkaloids

Alkaloids in the cardiovascular redox–inflammatory literature are represented mainly by berberine, discussed below.

Berberine is included because it provides a disease-specific connection among metabolic regulation, mitochondrial ROS, and NLRP3 inflammasome signaling. Experimental studies also associate berberine with AMPK, endothelial function, glucose and lipid metabolism, and inflammatory regulation [203,204,205,206,207].

As discussed in Section 3.3.5, berberine inhibited NLRP3 inflammasome activation in diabetic cardiomyopathy through regulation of the mTOR–mitochondrial ROS axis [106]. This sequence provides a mechanistic link between metabolic stress, mitochondrial redox disturbance, inflammasome activation, and cardiac injury. Its relevance has been demonstrated in a defined experimental setting and cannot yet be assumed across all cardiovascular conditions treated with berberine.

AMPK-related metabolic regulation may represent an upstream component of other reported effects [205,206]. Improved cellular energy handling can reduce mitochondrial stress and indirectly attenuate oxidative and inflammatory signaling. Berberine also has substantial intestinal exposure despite low systemic bioavailability [205,206]. Effects on intestinal signaling, the gut microbiota, bile-acid metabolism, and glucose or lipid handling may contribute to cardiovascular risk reduction without requiring high myocardial concentrations.

Human studies have focused mainly on surrogate cardiometabolic outcomes. A randomized trial in men with hyperlipidemia reported lower total cholesterol and a possible reduction in LDL cholesterol, while blood pressure, triglycerides, body mass index, and thromboxane A2 did not differ significantly from placebo [208]. Other trials and evidence syntheses have evaluated glucose regulation, postprandial lipids, adiposity, and related risk factors [203,204,209,210,211]. Product composition, dose, study population, and methodological quality vary substantially across this literature [203,204].

Improved glucose or lipid regulation may reduce cardiovascular risk without direct evidence of activity in myocardial AMPK, mitochondrial ROS, or NLRP3 signaling. Clinical pathway attribution requires evidence that the proposed target was engaged at the achieved exposure. Evidence for the prevention of major cardiovascular events remains limited.

Drug interactions also require attention. General reviews describe interactions between concentrated plant products and cardiovascular medicines through drug-metabolizing enzymes and transporters [212,213,214,215]. Repeated administration of berberine reduced CYP2D6, CYP2C9, and CYP3A4 activities in a human crossover study [216]. Clinical trials require authenticated products, systematic monitoring of adverse events, and prospective assessment of concomitant medications.

Berberine occupies an intermediate position in the mechanistic hierarchy of this review. The mTOR–mitochondrial ROS–NLRP3 sequence provides a strong disease-specific example, whereas much of the human evidence focuses on broader cardiometabolic improvement rather than direct engagement of the core pathways.

### 4.7. Phytoestrogens

Isoflavones and lignans are included primarily because they provide complementary evidence involving endothelial function, NO regulation, and host-dependent metabolism. Their direct relationship with the central redox–inflammatory pathways is less clearly established than that of the principal compounds discussed above.

Soy isoflavones can influence estrogen receptor signaling, eNOS expression, NO production, and antioxidant defense genes [217]. A Bayesian meta-analysis reported a modest overall signal for improved flow-mediated dilation after isoflavone-containing interventions [218]. The estimated response was greater for isolated isoflavones than for isoflavone-containing soy protein, although trials differed in formulation, participants, and baseline vascular status [218]. These results support an endothelial phenotype but do not demonstrate direct regulation of NOX, Nrf2, or NF-κB–NLRP3 signaling in treated participants.

Microbial metabolism adds substantial variability. Daidzein can be converted into equol by specific intestinal bacteria, while dietary lignans are transformed into enterodiol and enterolactone [219]. Only a subset of individuals consistently produces equol [219]. In a small randomized crossover study, soy isoflavones improved arterial stiffness in equol producers but not in nonproducers; isolated S-equol did not reproduce the response after a single dose in nonproducers [220].

A systematic review found that most analyses of equol-producer status were secondary and inadequately powered [221]. Some studies reported favorable changes in lipids, inflammation, or blood pressure among producers, whereas many reported null results [221]. Observational evidence has also linked higher enterolactone concentrations with lower cardiovascular and all-cause mortality, but residual confounding prevents a causal interpretation [222].

Phytoestrogens illustrate how sex, menopausal status, hormonal environment, baseline endothelial function, and microbial phenotype can alter the response to the same dietary exposure [217,218,219]. Their evidence supports the clinical relevance of endothelial and host–microbial factors but currently plays a complementary role in the pathway-centered argument of this review.

### 4.8. Integration According to Mechanistic Relevance

The compounds reviewed in Section 4 do not occupy equivalent positions within the central argument of this review. Quercetin and related flavonoids connect to NOX activity, Nrf2, eNOS/NO, and inflammatory signaling. Resveratrol links endothelial NO regulation and mitochondrial metabolism to inflammatory and remodeling responses. Curcumin converges on Nrf2, NF-κB, and NLRP3 signaling. Sulforaphane provides a comparatively well-defined Keap1–Nrf2 mechanism. Berberine offers a disease-specific connection among metabolic stress, mitochondrial ROS, and NLRP3 activation. The relative strength of pathway-level evidence across these compound classes is summarized in Figure 5.

Designation as a core compound reflects mechanistic relevance to the selected network, not proven clinical superiority. Even among these compounds, pathway dependence and achievable human exposure vary considerably. A compound may alter several downstream markers because one upstream disturbance has improved, without directly engaging every measured target.

Phenolic acids, carotenoids, and phytoestrogens occupy a supporting position. Their studies provide evidence involving endothelial function, oxidative biomarkers, lipid regulation, inflammation, or cardiovascular risk. Direct causal engagement of the core Nrf2, NOX, NF-κB–NLRP3, mitochondrial ROS, and eNOS pathways has been established less consistently. These compounds broaden the clinical context without defining the main mechanistic framework.

Pharmacokinetic differences further influence the strength of pathway interpretation. Polyphenols undergo extensive conjugation and microbial transformation [127,128,166,167,168,169,170,171,172,173,174]. Curcumin exposure varies by formulation [180,181,182]. Carotenoids require lipid-dependent intestinal absorption [186]. Sulforaphane depends on myrosinase-mediated conversion of the precursor [201,202]. Berberine may act through both intestinal and systemic processes [205,206]. Phytoestrogen responses are partly determined by microbial metabolite production [219,220].

The mechanistic evidence summarized above also differs in the proximity of the reported effect to a defined molecular target. To distinguish direct target engagement from pathway-level regulation or nonspecific antioxidant effects, Accordingly, Table 1 explicitly distinguishes direct chemical target engagement from pathway dependence or association and from nonspecific ROS scavenging, thereby avoiding attribution of a protein-level mechanism where direct molecular evidence is unavailable. It summarizes the dominant mechanistic interpretation for representative phytochemicals. Particular attention is given to electrophile-sensitive cysteine residues, because covalent modification of such residues provides substantially stronger evidence of proximal target engagement than changes in downstream ROS, cytokines, or pathway-associated proteins. Oridonin is included as a mechanistic comparator because direct covalent engagement of NLRP3 provides an instructive contrast with phytochemicals for which suppression of inflammasome activity has been demonstrated without equivalent evidence of direct NLRP3 binding.

These comparisons argue against treating ‘antioxidant activity’ as a single molecular mechanism. Direct stoichiometric scavenging of ROS is chemically plausible for several phytochemicals and readily demonstrable in cell-free systems, but it does not necessarily explain their biological activity at achievable in vivo concentrations. The strongest mechanistic evidence instead supports a spectrum ranging from direct electrophilic modification of defined sensor proteins, exemplified by Keap1 and NLRP3, to regulation of enzyme activity, protein–protein interactions, transcriptional programs, mitochondrial function, and upstream inflammatory signaling. Accordingly, a reduction in cellular ROS should be interpreted as a functional redox outcome unless direct chemical scavenging or modulation of a defined ROS-generating target has been demonstrated under biologically relevant exposure conditions.

To provide a common mechanistic reference for the compound-specific evidence discussed below, the principal components of the redox–inflammatory network are summarized in Table 2. Importantly, their cardiovascular effects are context-dependent and cannot be uniformly classified as protective or detrimental.

Against this mechanistic background, Table 3 compares representative phytochemical classes according to the pathways most strongly implicated by the available evidence. The table also places these mechanistic signals alongside the corresponding human evidence and major translational constraints.

## 5. Disease-Specific Cardiovascular Phenotypes

The pathways introduced in Section 3 recur across cardiovascular diseases, but their biological importance depends on the affected cell type, the initiating injury, and the disease stage. NOX-derived ROS and impaired eNOS/NO signaling are prominent in vascular dysfunction and hypertension, mitochondrial redox disturbance becomes especially important during ischemia–reperfusion and heart failure, and NLRP3 activation contributes differently to atherosclerotic inflammation, diabetic myocardial injury, and atrial remodeling [27,28,30,37,38,47,106,227,228].

The following subsections apply the core redox–inflammatory network to individual cardiovascular phenotypes. Each disease is evaluated based on its dominant pathological process, the position of the selected pathway within that process, the available phytochemical evidence, and the endpoint required to demonstrate disease-relevant benefit.

### 5.1. Atherosclerosis

Atherosclerosis develops through lipid retention, endothelial activation, leukocyte recruitment, inflammatory signaling, and progressive remodeling of the arterial wall [48]. Disturbed flow at susceptible vascular sites alters endothelial NO signaling and promotes expression of adhesion molecules. Retained and modified lipoproteins then activate macrophages and other vascular cells, generating additional oxidative and inflammatory signals [48].

NLRP3 is particularly relevant in lipid-rich lesions. As introduced in Section 3.3.2, cholesterol crystals activate the NLRP3 inflammasome in macrophages [47,93]; within advanced plaques, this activity is further sustained by hypoxia, mitochondrial dysfunction, oxidized lipids, altered macrophage metabolism, and impaired clearance of dying cells [47,91,94,224,229].

Early atherosclerosis is dominated by endothelial dysfunction, lipid retention, and leukocyte recruitment. Advanced lesions increasingly involve macrophage death, defective efferocytosis, necrotic core expansion, vascular smooth-muscle-cell remodeling, collagen turnover, and thrombogenic complications [48]. A reduction in circulating oxidative or inflammatory biomarkers cannot determine whether these plaque-level processes have changed.

Preclinical phytochemical studies describe effects on lipid handling, endothelial activation, macrophage inflammation, gut microbial metabolism, and lesion burden [230,231,232,233,234,235,236]. Examples include emodin, berry-derived interventions, saponins, *Alpinia zerumbet*, honey constituents, and naringenin [230,231,232,233,234,235,236]. The interventions and proposed mechanisms are highly heterogeneous and cannot be interpreted as evidence for a common antioxidant action.

Some studies more directly link a phytochemical response to the pathways selected in this review. MCL attenuated atherosclerosis by suppressing macrophage ferroptosis through KEAP1–NRF2 signaling [237]. β-Sitosterol reduced lesion development in ApoE-deficient mice in association with MAPK, Nrf2, and NLRP3 regulation [238]. These models provide pathway-specific hypotheses, but the measured pathway changes may still include downstream consequences of altered lipid metabolism or lesion burden.

Total lesion area is an incomplete measure of therapeutic relevance. Evaluation of macrophage burden, necrotic core size, collagen content, fibrous cap structure, vascular smooth-muscle-cell phenotype, efferocytosis, and thrombogenicity provides more information about plaque progression and stability. Studies of established lesions are particularly important because prevention of lesion initiation does not demonstrate regression or stabilization of advanced disease.

Human nutraceutical studies more commonly evaluate circulating lipids, blood pressure, inflammatory markers, or endothelial function than plaque composition [153,239]. These endpoints may indicate risk-factor improvement but cannot independently establish direct modification of plaque biology. Imaging of plaque burden or composition, measurement of active phytochemical metabolites, and clinical cardiovascular outcomes are needed before an anti-atherosclerotic effect can be distinguished from general cardiometabolic benefit.

### 5.2. Abdominal Aortic Aneurysm

Abdominal aortic aneurysm (AAA) is characterized by inflammatory-cell infiltration, vascular smooth-muscle-cell loss, extracellular-matrix degradation, protease activation, and progressive failure of aortic-wall integrity [240,241,242]. Mechanical stress, intraluminal thrombus, iron-related injury, endoplasmic-reticulum stress, and oxidative signaling interact during aneurysm expansion [240,242,243,244].

NOX enzymes provide regulated sources of ROS within the aneurysmal wall [72,227]. Persistent NOX activity may amplify vascular inflammation, matrix degradation, and smooth-muscle-cell dysfunction, although the roles of individual NOX isoforms vary across cell types and experimental models [72,227]. Iron accumulation and redox-active heme products may provide additional oxidative stimuli, particularly in the context of intraluminal thrombus [243].

The endogenous antioxidant response may also change with disease progression. A human study of male patients stratified by aneurysm diameter identified size-related differences among NRF2, HO-1, PON1, bilirubin, and inflammatory markers [71]. The authors interpreted the findings as evidence that chronically activated antioxidant responses may become inadequate or dysregulated as inflammation progresses [71]. Increased circulating NRF2 cannot be automatically equated with effective antioxidant protection, as it may reflect an attempted response to persistent tissue stress.

Compared with atherosclerosis, evidence for phytochemicals in AAA remains limited. Most available studies address oxidative stress, NOX activity, iron handling, endoplasmic-reticulum stress, inflammation, or mechanical signaling rather than testing chemically defined phytochemicals in longitudinal human interventions [227,240,241,242,243,244]. Evidence that a compound changes an antioxidant marker in an experimental model does not establish reduced aneurysm expansion or rupture risk.

Experimental AAA models reproduce selected inflammatory, proteolytic, or hemodynamic components of the human disease [240,241,242]. No single model fully represents the prolonged natural history, intraluminal thrombus, mechanical loading, heterogeneous wall structure, and rupture biology of human AAA. A reduction in maximal aortic diameter is more informative when accompanied by the preservation of elastin and collagen, smooth-muscle-cell survival, lower protease activity, and improved biomechanical integrity.

The clinically relevant outcomes are aneurysm growth, need for repair, rupture, and safety. Redox or inflammatory biomarkers can support a mechanism but cannot replace these outcomes. AAA remains a useful example of a disease in which apparent pathway activation may represent compensation for advanced tissue injury rather than reversal of the underlying pathology.

### 5.3. Hypertension

Hypertension involves renal, neural, endocrine, and vascular mechanisms. Within the vascular compartment, increased NOX activity, reduced NO bioavailability, eNOS uncoupling, endothelial dysfunction, and remodeling of resistance vessels contribute to elevated vascular tone and target-organ injury [37,38,45,79]. Nrf2-dependent defense may influence vascular redox balance, but its contribution varies across models, disease duration, and treatment contexts [65].

Human evidence indicates that some phytochemical-rich foods and isolated compounds can produce modest reductions in blood pressure. Systematic reviews and meta-analyses have evaluated flavonoid-rich fruits, edible algae, pomegranate, blueberries, and hibiscus [245,246,247,248,249,250]. Observational evidence also links dietary polyphenol intake with blood pressure and hypertension risk [251]. A recent cocoa extract trial extended this literature by examining incident hypertension rather than relying solely on short-term blood pressure change [252].

As reviewed in Section 4.1.1, quercetin supplementation has produced small, consistent reductions in blood pressure across meta-analyses [137,138]. Flavan-3-ol interventions have also been associated with modest improvements in blood pressure and endothelial function [152]. As discussed in Section 3.4.5 and Section 4.3, curcumin preparations may improve flow-mediated dilation, although effects on other vascular measurements remain variable [123].

A fall in blood pressure demonstrates a physiological response but does not identify its initiating mechanism. Renal sodium handling, sympathetic activity, weight change, arterial stiffness, endothelial function, and peripheral vascular resistance can all contribute. Improvement in flow-mediated dilation supports a vascular component but does not prove Nrf2 activation, NOX inhibition, or direct modulation of eNOS.

Mechanistic hypertension studies are most informative when standardized office or ambulatory blood pressure is assessed alongside endothelial function, arterial stiffness, biochemical exposure, dietary intake, body-weight change, and background medication. Food-based interventions and isolated compounds also require separate interpretation because potassium, sodium, energy intake, and other matrix components may influence the response.

Hypertension provides useful human evidence for a measurable cardiovascular phenotype, but it also demonstrates the limits of mechanistic inference from a single clinical endpoint. A pathway-specific claim requires evidence that the relevant redox or endothelial target changed at the exposure associated with the blood-pressure response.

### 5.4. Ischemia–Reperfusion Injury and Myocardial Infarction

Reperfusion restores oxygen and substrates to ischemic myocardium but also produces rapid metabolic and ionic changes. Mitochondrial ROS generation, calcium overload, mitochondrial permeability transition, microvascular injury, and multiple forms of cardiomyocyte death contribute to reperfusion injury [27,28,253]. NOX-derived ROS can interact with mitochondrial redox processes during ischemia and reperfusion [17].

The role of ROS is strongly time- and compartment-dependent. A large and sustained mitochondrial oxidative burst during reperfusion promotes protein and lipid oxidation, permeability transition, and cell death [28,29]. Brief and spatially controlled ROS signals also participate in endogenous cardioprotective responses, including ischemic preconditioning [28,29]. Complete suppression of reactive species could interfere with adaptive signaling while reducing injurious oxidation.

This duality changes the interpretation of phytochemical studies. Lowering total ROS, lipid peroxidation, or antioxidant enzyme activity after treatment does not show that a compound selectively inhibited the damaging reperfusion-associated burst. The ROS source, subcellular site of action, treatment timing, and relationship to mitochondrial permeability transition are at least as important as the overall magnitude of an antioxidant response.

The preclinical literature includes broad reviews of natural products and polyphenols, compound-specific studies, and investigations of NLRP3 or calcium-related pathways [254,255,256,257,258,259,260,261]. EGCG has reduced myocardial injury in meta-analysis of animal ischemia–reperfusion and infarction studies [144]. Curcumin pretreatment has also reduced experimental injury through pathways involving ferroptosis, autophagy, and apoptosis [262]. These findings demonstrate preclinical cardioprotection but do not establish clinical feasibility.

Pretreatment studies are most relevant to elective ischemia, planned cardiac procedures, or chronic preventive use. They cannot be transferred directly to an unexpected myocardial infarction, where treatment is usually initiated near or after reperfusion. A clinically relevant post-ischemic intervention must reach the myocardium rapidly enough to affect mitochondrial permeability transition, calcium-dependent injury, microvascular obstruction, inflammation, or cell death [253].

Variation in species, ischemia duration, reperfusion interval, anesthetic regimen, comorbidities, and background medication also contributes to poor translation [253,254,255,256,257,258,259,260,261]. Diabetes, aging, and concurrent cardiovascular drugs can modify endogenous cardioprotective pathways. Experimental programs require administrative schedules and models that mirror the intended clinical setting.

Infarct size remains an important endpoint, but clinical translation also requires evaluation of microvascular obstruction, ventricular remodeling, heart-failure development, and patient outcomes [253]. Ischemia–reperfusion provides the clearest example of why redox modulation must preserve adaptive signaling while limiting a temporally defined injurious process.

### 5.5. Heart Failure

Heart failure involves mitochondrial dysfunction, altered substrate utilization, neurohumoral activation, endothelial impairment, inflammation, fibrosis, and ventricular remodeling [30,31]. The relative contribution of each process varies with etiology, disease severity, and ejection-fraction phenotype. Heart failure with reduced, mildly reduced, and preserved ejection fraction is a clinically and biologically heterogeneous condition [263].

Mitochondrial dysfunction links several components of the selected mechanistic network. Impaired oxidative phosphorylation and altered mitochondrial membrane composition can reduce energetic efficiency and increase redox stress [30,31]. Mitochondrial injury may then promote inflammatory signaling, cardiomyocyte dysfunction, and adverse remodeling. These processes cannot be adequately represented by a circulating oxidative stress marker alone.

Most evidence for phytochemicals in heart failure remains mechanistic, preclinical, or based on small, heterogeneous human studies [31,264,265,266,267,268]. Reviews discuss polyphenols, EGCG, resveratrol, dietary interventions, and gut microbiota-related mechanisms [31,161,162,264,265,266,267,268]. Reported effects include changes in mitochondrial function, inflammation, fibrosis, ventricular remodeling, exercise-related outcomes, and selected biomarkers. Differences in compounds, models, populations, and endpoints prevent a unified estimate of benefit.

Resveratrol has been evaluated in cardiovascular disease and heart-failure populations, but clinical results remain variable and the studies are not sufficient to establish improved prognosis [161,162]. EGCG has extensive molecular and preclinical support, while direct evidence of heart failure in humans is limited [265]. Even coenzyme Q10, which has a larger heart-failure literature than many phytochemicals, retains uncertainty in pooled clinical evidence [269].

Clinical relevance requires a well-defined heart-failure phenotype. Results from pressure-overload, ischemic, toxin-induced, or metabolic models cannot be generalized automatically to ischemic HFrEF, nonischemic HFrEF, or HFpEF. A phytochemical intervention would also need to demonstrate incremental benefit alongside contemporary guideline-directed therapy [263].

Relevant outcomes include ventricular structure and function, congestion, exercise capacity, symptoms, quality of life, hospitalization, and mortality. Exposure and mechanism-matched biomarkers can explain how an effect occurred, but they cannot replace functional or clinical outcomes. Heart-failure research benefits from stratification by ejection fraction, etiology, disease severity, metabolic phenotype, and background treatment.

### 5.6. Diabetes-Associated Cardiovascular Disease

Diabetes produces interacting metabolic, vascular, and myocardial abnormalities. Hyperglycemia, altered lipid metabolism, mitochondrial dysfunction, oxidative stress, inflammation, and impaired NO signaling contribute to endothelial injury and diabetic cardiomyopathy [270,271,272,273,274,275,276,277]. These mechanisms coexist with changes in body weight, insulin resistance, blood pressure, renal function, and lipid concentrations.

Diabetic cardiomyopathy provides a setting in which mitochondrial stress, inflammatory activation, apoptosis, fibrosis, and contractile dysfunction can be assessed together [270,271,272,273,274,275,276,277]. Experimental models commonly report benefits of flavonoids and other bioactive compounds via mitochondrial, endoplasmic reticulum, inflammatory, and cell-death pathways [270,271,272,273,274,275,276,277]. Many studies use severe or untreated hyperglycemia, which may not represent contemporary treated diabetes.

As discussed in Section 3.3.5 and Section 4.6, berberine inhibited NLRP3 activation in diabetic cardiomyopathy by regulating the mTOR–mitochondrial ROS axis [106]. This mechanism does not establish NLRP3 inhibition as the primary explanation for all cardiometabolic effects of berberine, and diabetes is one of the clearest disease-specific settings in which this pathway has been tested.

Human evidence more frequently concerns cardiometabolic risk factors than direct myocardial effects. In the TOSCA.IT population, polyphenol intake was examined in relation to cardiovascular risk factors among individuals with type 2 diabetes [278]. Such findings can support an association with cardiometabolic health but cannot determine whether a phytochemical directly protects the myocardium or vascular wall.

Improved glycemic control, weight, or lipid concentrations can indirectly reduce cardiovascular risk. A direct cardiovascular claim requires vascular or myocardial improvement that remains interpretable after accounting for these metabolic changes. Measures of endothelial function, ventricular structure, diastolic or systolic function, exercise capacity, and cardiac biomarkers can help distinguish direct and indirect effects.

Mechanism-matched studies may be most informative in patients who retain the proposed abnormality, such as endothelial dysfunction, elevated inflammatory activity, or impaired redox regulation. Enrollment based solely on a diabetes diagnosis may combine patients with different dominant mechanisms, reducing the observed average response.

### 5.7. Atrial Fibrillation

Atrial fibrillation (AF) differs from primarily vascular phenotypes because oxidative and inflammatory signaling contribute to the formation of an arrhythmogenic myocardial substrate. Mitochondrial dysfunction, altered calcium handling, oxidative damage, inflammation, cardiomyocyte dysfunction, and fibrosis interact during atrial remodeling [279,280,281,282,283]. Persistent AF can also contribute to ventricular dysfunction and AF-induced cardiomyopathy [284].

Cardiomyocyte NLRP3 activation provides a direct connection to the core pathways of this review. Experimental enhancement of cardiomyocyte NLRP3 signaling promoted electrical remodeling and AF susceptibility, while inhibition attenuated the arrhythmogenic phenotype [228]. The study localized inflammasome activity within atrial cardiomyocytes and showed that inflammation is not limited to circulating immune cells [228].

The importance of each pathway changes with substrate maturity. Oxidative and inflammasome signaling may contribute to early electrical and cellular remodeling, whereas established atrial fibrosis creates a more persistent structural substrate [281,282,285]. An intervention that reduces inflammatory signaling during early remodeling may not reverse advanced fibrosis or long-standing AF.

Quercetin has been discussed in relation to oxidative, inflammatory, ion-channel, and remodeling mechanisms relevant to cardiac arrhythmia [225]. The available evidence remains predominantly experimental and review-based. It does not demonstrate that quercetin or any other phytochemical reduces the incidence, burden, or recurrence of clinical AF.

The literature considered here provides mechanistic hypotheses but no sufficient basis for claims of phytochemical rhythm control [228,279,280,281,282,283,284,285,286]. Relevant clinical outcomes include incident AF, objectively measured AF burden, recurrence after cardioversion or ablation, atrial structural remodeling, hospitalization, stroke, and safety. Systemic oxidative or inflammatory biomarkers cannot independently demonstrate modification of the atrial substrate.

AF studies also need to distinguish incident, paroxysmal, persistent, postoperative, and obesity-associated phenotypes. Differences in substrate, comorbidity, fibrosis, and background treatment may alter responsiveness to redox-directed intervention. Interaction with anticoagulant and antiarrhythmic therapy requires prospective safety assessment if concentrated phytochemical products are tested clinically.

### 5.8. Disease Phenotype and Stage as Determinants of Response

The same redox–inflammatory pathway can have different meanings across cardiovascular diseases. NLRP3 participates in macrophage-driven plaque inflammation, metabolic myocardial injury, and atrial remodeling [47,93,106,228]. Mitochondrial ROS contribute to both reperfusion injury and adaptive cardioprotective signaling [28,29]. Nrf2 activation may support cellular defense, yet elevated NRF2-associated markers in advanced AAA can reflect an inadequate response to continuing tissue stress [71].

Disease stage adds another level of variation. A pathway may contribute to disease initiation, remain active during progression, become compensatory in established disease, or lose importance after irreversible structural remodeling. Lesion initiation and plaque instability, early and advanced AAA, acute reperfusion and chronic remodeling, and early electrical versus fixed fibrotic atrial substrates require different endpoints and treatment windows.

A disease-relevant phytochemical study must align five elements: the active compound or metabolite, achievable tissue exposure, the targeted pathway, the cardiovascular phenotype, and the stage at which the pathway remains modifiable. Pathway engagement gains clinical meaning only when it is linked to an endpoint that represents the corresponding disease process.

This framework also explains why the same intervention can produce different results across models or patient populations. A weak average clinical response may reflect mechanistic heterogeneity rather than the absence of all biological activity. Strong experimental effects can disappear when the selected pathway is no longer dominant, the human exposure is inadequate, or the endpoint does not capture the targeted process. Disease phenotype and stage must be incorporated into the study design before subgroup results are known.

## 6. Determinants of Human Exposure and Biological Response

The molecular mechanism attributed to a phytochemical is meaningful only if the relevant parent compound or metabolite reaches the target tissue at an adequate concentration and for an appropriate duration. Exposure is therefore part of the mechanism rather than a technical consideration applied after efficacy has been observed [128,287,288].

### 6.1. Parent Compounds, Conjugates, and Circulating Metabolites

The disparity between concentrations used in experimental studies and those achieved in humans remains one of the major obstacles to translating phytochemical biology into cardiovascular therapeutics [128,287,288]. An administered dose cannot be assumed to represent biologically active exposure. Absorption, intestinal and hepatic metabolism, conjugation, tissue distribution, food matrix, formulation, and microbial biotransformation collectively determine which molecular species ultimately reach the circulation and target tissues [128,287,288]. These considerations are particularly important when mechanistic effects observed at micromolar concentrations in vitro are invoked to explain responses to dietary or supplemental exposure [128,287,288].

Quercetin illustrates the complexity that can exist within a single compound family. Absorption depends on glycoside structure, food matrix, intestinal transformation, and host metabolism, while circulating exposure is dominated by conjugated metabolites rather than the aglycone commonly used in cell experiments [128,132]. Nominal dose alone is therefore an incomplete measure of the molecular species and concentrations encountered by cardiovascular tissues [128,132].

Resveratrol provides a clear distinction between absorption and systemic availability. It is absorbed but rapidly conjugated in the intestine and liver, resulting in low circulating concentrations of the unchanged parent compound [166]. Mechanistic interpretation of oral resveratrol must consequently consider glucuronidation and sulfation conjugates, potential tissue deconjugation, microbial products, and the possibility that in vitro studies of parent resveratrol do not recapitulate human exposure [127,166,289,290,291,292].

Curcumin is among the most prominent examples of formulation-dependent exposure. Conventional oral curcumin generally produces low systemic concentrations, whereas piperine-containing, phospholipid-based, micellar, and nanoparticle formulations can markedly change absorption and disposition [180,181,182]. These technologies improve pharmacological feasibility but yield distinct interventions; findings from enhanced formulations should not be automatically transferred to native curcumin or dietary turmeric [180,181,182].

### 6.2. Bioavailability and Formulation

For some phytochemicals, bioavailability is determined before absorption begins. Sulforaphane exposure depends on conversion of glucoraphanin by myrosinase, an enzyme whose activity is markedly reduced by thermal processing [196,201,202]. Consequently, raw and cooked broccoli can provide substantially different systemic sulforaphane exposure despite containing the same precursor pathway [196,201,202]. Food preparation is therefore not a trivial methodological variable but rather part of the pharmacokinetic determinants of biological activity.

These observations also challenge the assumption that greater systemic exposure should invariably produce greater cardiovascular benefit. The hormetic model offers a biologically plausible alternative, in which relatively low phytochemical exposure produces a controlled electrophilic or oxidative stimulus that activates endogenous adaptive programs, including Nrf2-dependent cytoprotection [293]. Under this model, the therapeutic objective is not maximal antioxidant exposure but an appropriate biological response within a dose range that preserves physiological redox signaling [293]. This concept is consistent with the context-dependent nature of Nrf2 biology discussed in Section 3.5 and argues against simple linear extrapolation from dose to antioxidant efficacy.

Bioavailability is not merely a technical obstacle; it is part of the mechanism. The relevant question is which molecular species reaches the target tissue, at what concentration, and for how long. The exposure must also be sufficient to engage the proposed redox–inflammatory pathway without disrupting normal redox signaling [127]. Defining this exposure–target relationship is essential for reproducible cardiovascular benefit.

Minimum reporting should include source material, extraction and standardization, batch composition, stability, dosing with respect to food, parent and metabolite exposure, adherence, concomitant medication, and prespecified safety surveillance [127]. These variables are necessary to reproduce the intervention and to determine whether discrepant trials tested the same pharmacological entity [127].

### 6.3. Food Matrix and Processing

Food-based exposures cannot be interpreted as interventions involving purified compounds. Processing, macronutrient composition, co-ingestion, and the structure of the food matrix alter release, absorption, and metabolism of polyphenols, while products such as chocolate and wine contain constituents that can reinforce or oppose the effects attributed to a single phytochemical [156,288,291,292,294,295,296].

This distinction is especially important when mechanistic studies use resveratrol, cocoa flavanols, or isolated anthocyanins to explain results obtained with wine, chocolate, berries, or mixed dietary patterns. The whole-food result should be attributed to the tested matrix unless exposure to the proposed active species and target engagement are measured directly [156,288,291,292,294,295,296].

### 6.4. Gut Microbiome and Metabotypes

The network becomes more complex when the compound consumed is not the principal molecular species reaching the circulation. Many polyphenols undergo extensive intestinal, microbial, and hepatic metabolism, generating metabolites with biological properties distinct from those of their dietary precursors [297].

Ellagitannins provide an instructive example. Their microbial metabolism generates urolithins, and urolithin B has been reported to activate Nrf2-associated signaling and reduce myocardial injury in experimental ischemia–reperfusion [298]. These findings establish the biological activity of a microbiota-derived metabolite, although they do not demonstrate that urolithin B alone mediates the cardiovascular effects of dietary ellagitannins.

Interindividual differences in microbial metabolism may consequently contribute to variability in phytochemical trials [299]. Identical intake does not necessarily produce equivalent systemic exposure when participants differ in their capacity to generate bioactive metabolites [299]. For compounds dependent on microbial transformation, circulating metabolite profiles—and potentially microbial metabotypes—may therefore be more informative than administered dose alone [299]. Future trials should incorporate metabolite measurements where feasible, rather than assuming biological exposure based on intake.

Metabotype-aware analysis offers a practical route to test microbiome dependence. Participants can be classified by their ability to generate urolithins, equol, or other microbial products, while targeted metabolomics quantifies the actual circulating species [127,219,290,300]. This approach can distinguish failure of the parent intervention from failure to generate the bioactive metabolite and may explain part of the heterogeneity obscured in conventional intention-to-treat averages [127,219,290,300].

### 6.5. Epigenetic and Persistent Regulatory Effects

Phytochemicals have also been associated with changes in DNA methylation, histone regulation, and non-coding RNAs affecting Nrf2, NF-κB, NLRP3, and related pathways [301]. Such mechanisms are potentially important because they could modify cellular responses beyond the period of measurable exposure to the parent compound.

MicroRNA-dependent regulation provides one example. Nrf2-associated protection during myocardial ischemia–reperfusion has been linked to miR-29a-3p signaling [302]. However, an accompanying change in microRNA expression does not establish mechanistic mediation. Causality requires demonstration that selective manipulation of the proposed epigenetic regulator modifies both pathway activity and the cardiovascular phenotype [126,302].

Epigenetic effects should therefore be considered a potentially important layer between phytochemical exposure and sustained cellular adaptation, while mechanistic claims remain dependent on functional validation [126,301,302].

### 6.6. Drug Interactions and Safety

The proposed activity of phytochemicals across several pathways also creates potential for clinically relevant drug interactions [212]. This is particularly important in cardiovascular disease, where phytochemical supplements are commonly consumed alongside lipid-lowering, antihypertensive, antithrombotic, and glucose-lowering therapies [212].

Interactions may be pharmacokinetic or pharmacodynamic. Dietary flavonoids can interfere with CYP enzymes and membrane transporters involved in drug disposition, including CYP3A4 and organic anion-transporting polypeptides, potentially altering statin exposure and susceptibility to muscle toxicity [213]. Interactions involving phytochemicals used alongside antiplatelet agents have also been described [213].

Resveratrol exemplifies potential pharmacodynamic overlap, as it exhibits antiplatelet activity [214]. This creates a plausible basis for additive platelet inhibition during concomitant antiplatelet therapy, although evidence of clinically meaningful excess bleeding remains insufficient [214]. The distinction between mechanistic plausibility and demonstrated clinical harm should therefore be maintained.

These considerations are especially relevant to concentrated extracts and enhanced-bioavailability preparations, for which systemic exposure may substantially exceed that achieved through ordinary dietary intake [215]. The assumption that naturally derived compounds are intrinsically safe is therefore pharmacologically untenable; safety depends on dose, exposure, metabolism, concomitant treatment, and patient susceptibility [215].

## 7. From Preclinical Activity to Clinical Evidence

The principal limitation in phytochemical cardiovascular research is no longer the shortage of mechanistic observations, but the difficulty of determining which of them remains relevant under human physiological conditions. Cell-based studies have been indispensable for identifying redox-sensitive pathways, yet the experimental environment itself can substantially influence the phenotype being measured [303]. Standard culture conditions expose cells to oxygen tensions that exceed those of most tissues and lack the extracellular antioxidant buffering provided by plasma [303]. Consequently, an apparent protective effect may partly reflect the correction of culture-induced oxidative stress rather than the modulation of a mechanism that operates to the same extent in vivo.

### 7.1. Hierarchy of Evidence

Animal models provide the essential intermediate step between molecular mechanism and human disease, allowing assessment of tissue injury, remodeling, vascular function, and whole-organ physiology [304]. Their limitations, however, are particularly relevant to cardiovascular redox biology. Species differences in lipid metabolism, coronary physiology, thrombosis, and the expression of redox enzymes can alter both disease mechanisms and treatment responses [304]. The absence of NOX5 from conventional rodent models is a notable example: a potentially important component of human vascular oxidative stress is simply not represented in much of the experimental literature [304].

Human evidence remains considerably less mature. Available trials are generally small and of limited duration, with substantial heterogeneity in population, dose, formulation, and background treatment [305]. Interpretation is further complicated by reliance on oxidative stress biomarkers such as F2-isoprostanes, oxidized LDL, and malondialdehyde, whose analytical performance and reproducibility vary across assays and laboratories [305]. A change in an oxidative biomarker may support biological activity, but it cannot substitute for evidence of target engagement or improvement in cardiovascular function.

Evidence levels answer different questions and should not be treated as interchangeable. Cell experiments identify molecular plausibility, loss-of-function studies test dependence, animal models evaluate integrated physiology, pharmacokinetic studies establish attainable exposure, and randomized trials estimate functional or clinical effects. A mechanistic claim becomes stronger only when these levels form a coherent chain rather than when many low-level observations point in the same direction [126,129,130,239].

### 7.2. Dose and Exposure Mismatch

Concentration is an equally important concern. Many mechanistic studies employ phytochemicals at 10–100 μM, whereas circulating concentrations of the parent compounds after oral intake are generally much lower [15,128,166,180,303,306]. At high concentrations, some polyphenols can undergo auto-oxidation, generating hydrogen peroxide in culture media and producing pro-oxidant effects that further complicate interpretation [15,303,306]. Thus, mechanistic plausibility requires not only the demonstration of pathway modulation but also evidence that the responsible molecular species and concentrations are achievable in vivo.

Dose conversion based only on body weight does not resolve this problem. Route, formulation, protein binding, conjugation, tissue distribution, and the duration of exposure determine whether cardiovascular cells encounter the same molecular species studied experimentally [128,287,288,289,290,291,292,294,295,307,308,309,310,311,312]. Studies should therefore compare free and total concentrations of parent compounds and metabolites with the concentration–response range of the proposed mechanism.

### 7.3. Target Engagement and Biomarkers

A rigorous translational framework should establish four levels of evidence: biologically relevant exposure, molecular target engagement, functional cardiovascular response, and clinical benefit [129]. Pharmacokinetic studies should demonstrate that the active parent compound or its metabolite achieves the required exposure. Pathway-specific measurements should confirm that the proposed target is engaged. Validated vascular or cardiac endpoints should then be used to establish physiological relevance, followed by well-designed clinical studies that assess patient benefit [129].

Target engagement should be as specific as the mechanistic claim. Increased expression of an Nrf2-regulated gene may support pathway activation, whereas a claim of NOX inhibition requires assessment of the relevant isoform or assembly process, and a claim of inflammasome inhibition should extend beyond NLRP3 abundance to caspase-1 activity, cytokine maturation, or gasdermin D cleavage [42,43,44,45,46,47,51,60,87,99,223,224,229,313,314]. Oxidative biomarkers remain useful, but they should be interpreted as complementary measures rather than substitutes for pathway-specific evidence.

### 7.4. Clinical Endpoints

A practical evidence hierarchy for future reviews and trials is therefore as follows: chemical identity and formulation; human pharmacokinetics; pathway-specific target engagement; validated vascular or myocardial function; and clinical outcomes [126,129,239]. Studies that reach only the first two levels establish exposure, those that reach target engagement establish pharmacological activity, and only studies showing functional or clinical benefit support cardioprotective relevance [126,129,239].

Functional endpoints should also align with the disease and the proposed mechanism. FMD can test NO-dependent conduit-artery function, ambulatory blood pressure can quantify hemodynamic response, cardiac imaging can evaluate remodeling, and exercise capacity can assess integrated heart-failure physiology. None is equivalent to fewer myocardial infarctions, strokes, hospitalizations, or deaths, so surrogate and clinical outcome claims should remain separate [19,129,130,239,315].

### 7.5. Trial Design and Standardization

This sequence also clarifies why both positive and negative trials can otherwise be difficult to interpret. Without evidence of exposure and target engagement, failure to improve a clinical endpoint cannot distinguish an ineffective mechanism from inadequate delivery of the intervention [130]. Conversely, changes in Nrf2, inflammatory mediators, or oxidative biomarkers without accompanying functional or clinical improvement establish biological activity, but not cardioprotection [130]. Closing this mechanistic–pharmacokinetic–clinical continuum is therefore the central requirement for moving phytochemical cardiovascular research from experimental plausibility to therapeutic credibility.

Future trials should distinguish chemically distinct formulations, prespecify the background diet and cardiovascular therapy, and report adherence, adverse events, parent-compound and metabolite exposures, and assay performance. Negative trials remain informative when exposure and target engagement are demonstrated; without these measurements, a negative outcome cannot distinguish failure of the intervention from failure to test the proposed mechanism [127,129,130,239,296].

The distinction between biological activity and clinically relevant exposure is particularly important when interpreting the available human studies. Table 4 considers the principal clinical signals together with the pharmacokinetic and formulation-related limitations that determine how confidently these findings can be translated.

## 8. Precision Phytochemical Intervention and Future Directions

### 8.1. Redox and Inflammatory Endotypes

Dose alone is unlikely to explain the variable response to phytochemicals. Patients with the same diagnosis may differ in the main source of ROS, inflammatory activity, endothelial NO bioavailability, metabolic status, and ability to produce bioactive phytochemical metabolites [300]. These differences support a more selective, mechanism-based approach instead of uniform supplementation.

A useful framework is provided by cardiovascular endotyping, in which patients are characterized according to the biological pathways most active within an otherwise heterogeneous clinical phenotype [316]. Applied to phytochemicals, this would mean selecting an intervention because its pharmacology corresponds to a demonstrable abnormality in the patient, not because the compound has broadly described “antioxidant” properties. Impaired endothelial function, excessive lipid oxidation, persistent inflammatory activation, or an altered Nrf2-responsive stress profile could, in principle, define different treatment-enriched populations [300]. The same biological abnormality used for selection should then be followed during treatment to establish whether the proposed target has actually been modified.

### 8.2. Biomarker-Guided Patient Selection

Multi-omics approaches may refine this strategy by identifying combinations of metabolic, inflammatory, and redox features that are not captured by conventional clinical phenotyping [300]. Their value, however, will depend on whether the resulting molecular signatures identify reproducible biological states and predict treatment response. Unsupervised molecular associations without prospective validation risk adding another layer of complexity without improving therapeutic selection. The objective is not an increasingly detailed molecular description, but the identification of actionable biology.

### 8.3. Timing and Disease Stage

Mechanism-matched selection should also account for disease timing. A redox intervention may preserve endothelial function or limit early inflammatory amplification before irreversible structural injury develops, yet provide little benefit after advanced fibrosis, plaque necrosis, or atrial substrate maturation. Trial eligibility and endpoints should therefore reflect whether the targeted process is causal, compensatory, or secondary at the proposed treatment stage [153,161,162,165,230,235,236,249,250,268].

### 8.4. Formulation and Combination Strategies

This approach also requires a clear therapeutic position. Phytochemicals should be evaluated as adjuncts to evidence-based cardiovascular treatment, not as alternatives to therapies with established effects on morbidity and mortality. This is particularly important because the patients most likely to receive phytochemical supplements are often already exposed to statins, antiplatelet or anticoagulant therapy, antihypertensive agents, and glucose-lowering drugs. Pharmacokinetic and pharmacodynamic interactions should therefore be incorporated prospectively into trial design rather than considered only after safety signals emerge.

Sex and age should be prespecified as biological variables rather than treated solely as demographic descriptors. They can influence vascular phenotype, metabolism, parent-compound and metabolite exposure, and treatment response [317]. Trials should therefore report sex-specific enrollment and analyses, age distribution, menopausal status when relevant, and possible interactions with the selected endotype [317].

### 8.5. Priorities for Future Research

Future clinical studies should be more selective and stricter in their pharmacological design. Investigators should use a chemically characterized, standardized intervention and confirm systemic exposure to the relevant parent compound or metabolite. The study population should be enriched for the targeted pathway, target engagement should be prespecified, and molecular effects should be linked to validated vascular, myocardial, or clinical outcomes. Each step answers a different question. Without exposure data, a negative trial cannot distinguish a failed mechanism from inadequate delivery. Without target-engagement data, it cannot confirm that the biological hypothesis was tested. Finally, target engagement without functional or clinical benefit shows pharmacological activity, not cardioprotection.

A hypothetical pilot trial illustrates the approach. Patients with diabetic cardiomyopathy could be enrolled only when a prespecified inflammatory–redox endotype is present, such as elevated IL-1β-related activity together with measurable myocardial dysfunction. A standardized berberine formulation could then be evaluated using parent compound and metabolite measurements, a predefined NLRP3/caspase-1/IL-1β target-engagement panel, a matched cardiac or endothelial functional endpoint, stable background therapy, and prospective interaction monitoring [106,209]. This example is a trial-design model, not a clinical treatment recommendation.

Precision phytotherapy is best regarded as a mechanism-guided experimental strategy rather than personalized supplement selection. Its clinical value will depend on whether prospectively defined molecular selection improves treatment consistency beyond unselected supplementation. Figure 6 summarizes the translational sequence from source and formulation through target engagement to biomarker-guided intervention [300].

The translational sequence illustrated in Figure 6 also identifies several recurring points at which otherwise promising phytochemical interventions may fail to generate interpretable clinical evidence. Table 5 translates these limitations into practical priorities for the design and reporting of future studies.

## 9. Limitations of the Review

This article is a narrative review informed by a structured PubMed search, not a systematic review or meta-analysis. Study selection and interpretation remain subject to author judgment, and the search may not capture every relevant compound, disease model, formulation, or non-PubMed source. The marked heterogeneity of interventions and endpoints also prevents pooled estimates of cardiovascular efficacy. The evidence base was updated through August 2026, so later studies are not included.

The review emphasizes redox–inflammatory mechanisms in selected vascular and myocardial phenotypes. Stroke and peripheral artery disease were not evaluated as separate disease sections, and sex- and age-specific effects were inconsistently reported in the underlying literature. These scope choices limit generalization across the full spectrum of cardiovascular disease. Dedicated systematic reviews and prospectively stratified clinical studies are needed to test the framework in those populations.

The breadth of the field also creates classification problems. Polyphenols, flavonoids, stilbenes, isoflavones, lignans, food extracts, and microbial metabolites are not mutually exclusive categories, and studies often use chemically different preparations under the same compound name. The present organization reduces this ambiguity but cannot eliminate variation in nomenclature, product composition, or analytical characterization.

A second limitation concerns inference across evidence levels. Cell and animal studies were used to identify plausible pathways, whereas human trials were used to evaluate exposure and functional effects. The review does not treat pathway changes in experimental systems as proof of clinical cardioprotection, but selective reporting and publication bias may still overrepresent favorable mechanistic results. The literature search was structured but narrative, so the review does not provide pooled effect estimates or a formal risk-of-bias assessment for every intervention.

Finally, the reference base is necessarily heterogeneous in recency and design. Foundational studies were retained where they established mechanisms, and recent reviews were used to map rapidly developing topics. This strategy supports broad mechanistic synthesis but means that confidence is higher for general pathway biology than for the efficacy of any specific phytochemical product.

## 10. Conclusions

The accumulated evidence argues against interpreting phytochemicals primarily as exogenous scavengers of reactive oxygen species. At concentrations relevant to human exposure, their more plausible cardiovascular actions involve modulating endogenous systems that govern how cells generate, sense, and respond to oxidative and inflammatory stress. Nrf2-dependent stress adaptation, NOX-derived ROS, mitochondrial function, NF-κB and NLRP3 signaling, and endothelial eNOS/NO homeostasis are therefore better viewed as components of an interconnected biological system than as independent targets.

The strength of this framework lies equally in recognizing its boundaries. Not all ROS are pathological, not all NOX activity is detrimental, and not all Nrf2 activation is necessarily beneficial. NOX4 can support adaptive H_2_O_2_-dependent signaling; transient ROS generation participates in ischemic preconditioning; and the biological consequences of sustained Nrf2 activation differ fundamentally from those of a transient, appropriately scaled stress response. Cardiovascular redox biology is therefore governed by source, compartment, magnitude, timing, and disease context. Any therapeutic model based simply on increasing “antioxidant capacity” is unlikely to capture this complexity. Importantly, the term “antioxidant” should not be interpreted as implying a uniform chemical mechanism: cardiovascular phytochemicals range from compounds with demonstrable covalent engagement of defined redox-sensitive proteins to agents whose effects are supported primarily at the level of pathway regulation, with direct stoichiometric ROS scavenging representing a distinct and not necessarily dominant mechanism in vivo.

The main challenge is no longer finding additional compounds with antioxidant activity; it is testing the most credible candidates rigorously in humans. Chemistry, pharmacokinetics, mechanism, patient selection, and outcome assessment should therefore be planned as connected parts of the same study.

The most credible future for phytochemical cardioprotection is neither broad supplementation nor replacement of established therapy. It is the development of chemically defined mechanism-matched adjunctive interventions for biologically selected patients. Whether phytochemicals can meet this standard remains uncertain, but this framework provides a clear way to test the question.

## Figures and Tables

**Figure 1:**
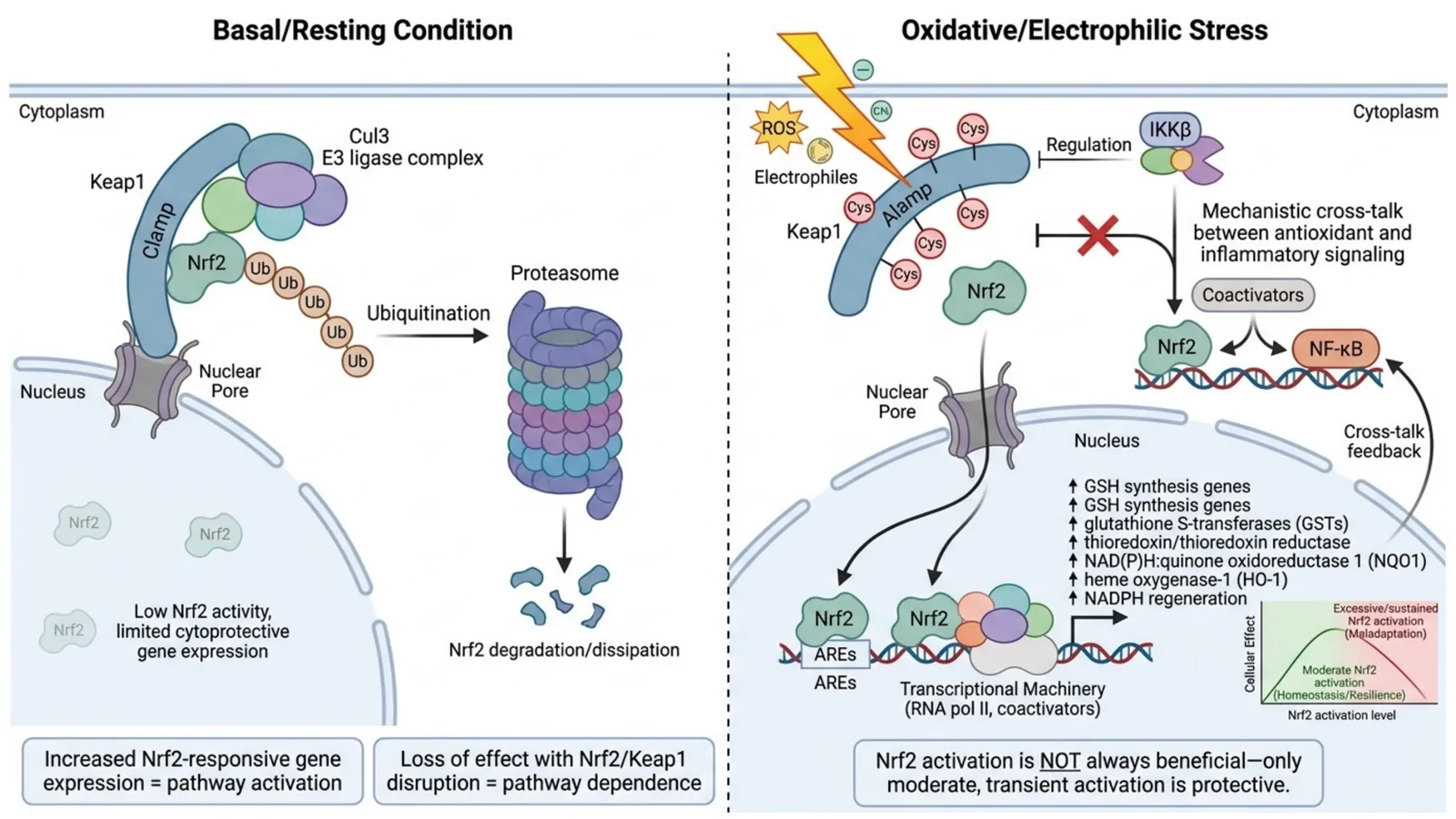
Keap1–Nrf2–ARE signaling and its functional crosstalk with NF-κB. Under basal conditions, Keap1 functions as a substrate adaptor for the Cul3 E3 ubiquitin–ligase complex, promoting continuous ubiquitination and proteasomal degradation of Nrf2. Oxidative or electrophilic stress modifies reactive Keap1 cysteine residues, impairing efficient Nrf2 degradation and allowing newly synthesized Nrf2 to accumulate, translocate to the nucleus, and activate ARE-dependent transcription. Nrf2 subsequently induces cytoprotective programs involving glutathione synthesis, GSTs, HO-1, NQO1, thioredoxin-dependent defenses, and NADPH regeneration. Functional crosstalk with NF-κB occurs at several levels, including Keap1–IKKβ regulation and competition for transcriptional coactivators. Increased Nrf2-responsive gene expression indicates pathway activation, whereas loss of an intervention effect following Nrf2/Keap1 perturbation provides stronger evidence of pathway dependence. The figure also emphasizes the redox-hormesis concept: moderate, transient Nrf2 activation may support adaptive stress resistance, whereas persistent dysregulated activation should not automatically be interpreted as beneficial. Created by Urbanowicz T on 24 August 2026 with www.figurelabs.ai (http://www.figurelabs.ai/) (ID: FL-PUB-20260824-01Z84W).

**Figure 2:**
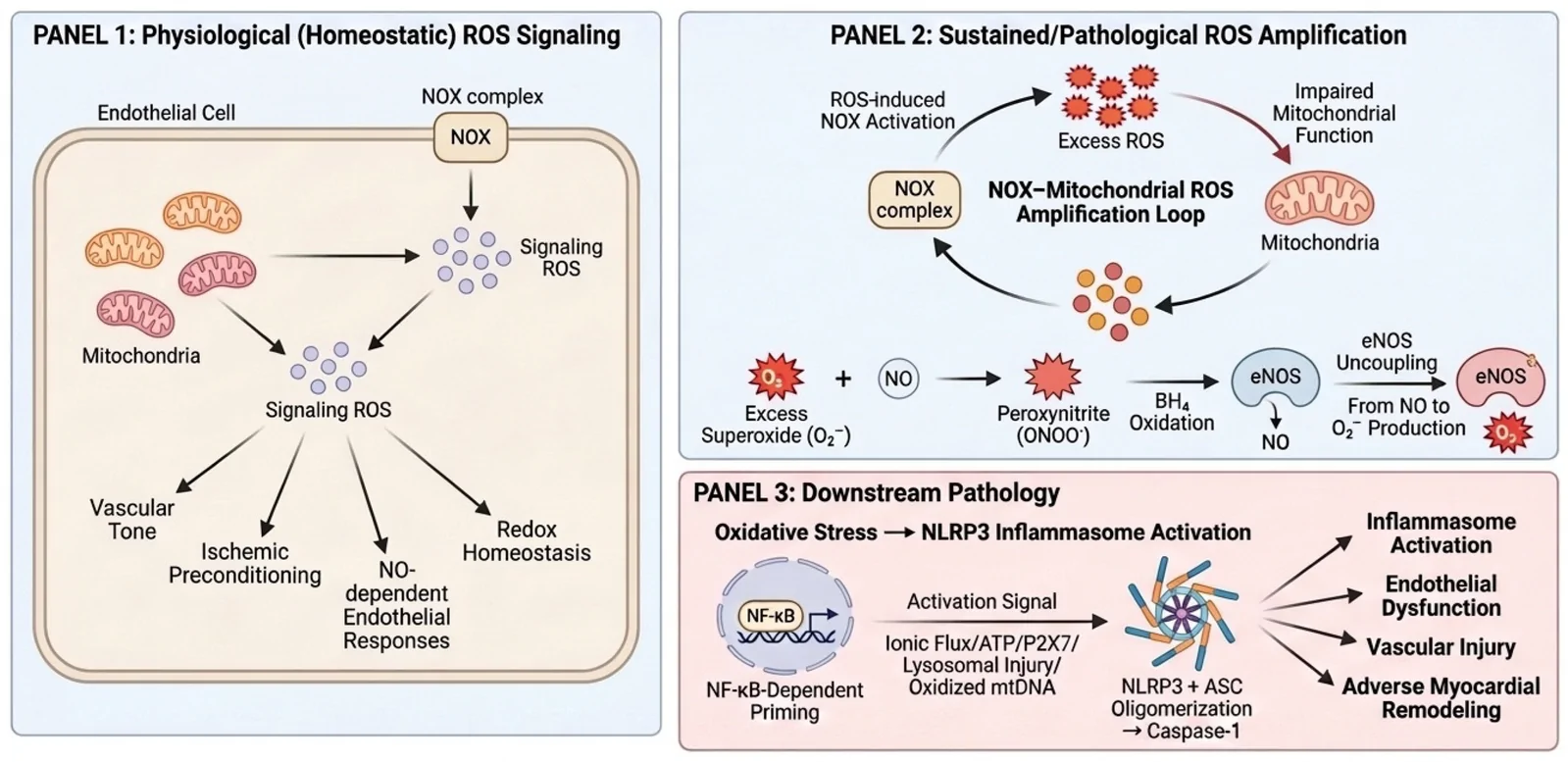
Physiological redox signaling versus pathological ROS amplification, showing NOX–mitochondrial crosstalk, the coupled/uncoupled eNOS transition, and the two-signal (priming plus activation) requirement for NLRP3 assembly (see Section 3.2 and Section 3.3). Created by Urbanowicz T on 24 August 2026 with www.figurelabs.ai (http://www.figurelabs.ai/) (ID: FL-PUB-20260824-OLCY59).

**Figure 3:**
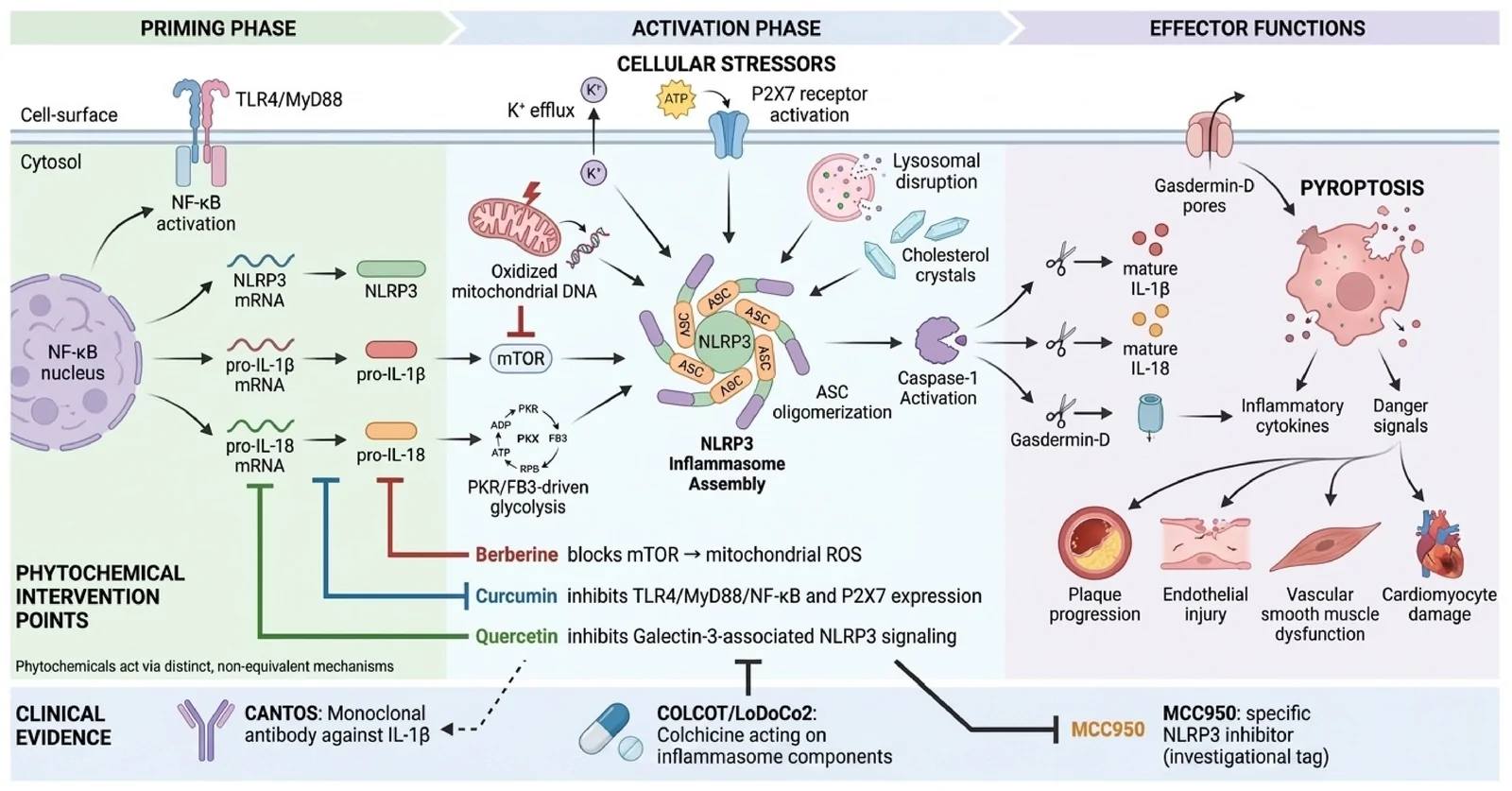
NLRP3 inflammasome priming, activation, and gasdermin D-mediated pyroptosis, mapped to representative phytochemical entry points (berberine, curcumin, quercetin) and to clinical trials validating inflammation as a cardiovascular target (CANTOS, COLCOT, LoDoCo2) alongside the selective comparator MCC950 (see Section 3.3). Created by Urbanowicz T on 24 August 2026 with www.figurelabs.ai (http://www.figurelabs.ai/) (ID: FL-PUB-20260824-8YOUWZ).

**Figure 4:**
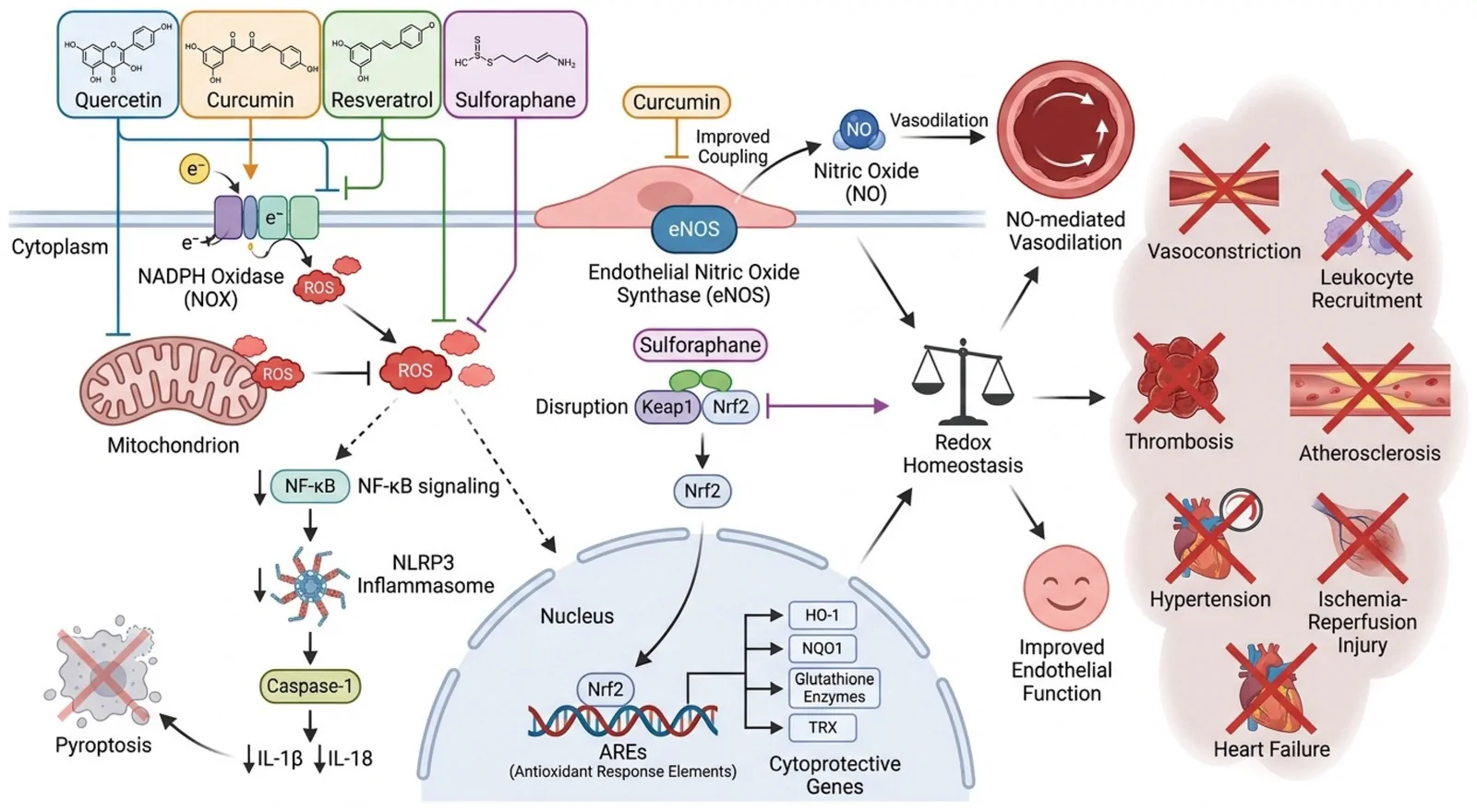
Proposed phytochemical modulation of cardiovascular redox–inflammatory signaling. Blue dashed arrows indicate proposed phytochemical actions, including inhibition of NOX-dependent ROS production, preservation of coupled eNOS and NO signaling, and activation of the Keap1–Nrf2 pathway. Green arrows indicate the protective route from coupled eNOS to NO-mediated vasodilation, thereby preserving vascular homeostasis. Red arrows show the pathological cascade from NOX enzymes, uncoupled eNOS, and mitochondria to excessive ROS, followed by NF-κB activation, NLRP3 inflammasome assembly, caspase-1 activation, IL-1β/IL-18 maturation, and pyroptosis. T-bars indicate inhibition, whereas arrowheads indicate activation or downstream signaling. Created by Urbanowicz T on 24 August 2026 with www.figurelabs.ai (http://www.figurelabs.ai/) (ID: FL-PUB-20260824-8YOUWZ).

**Figure 5:**
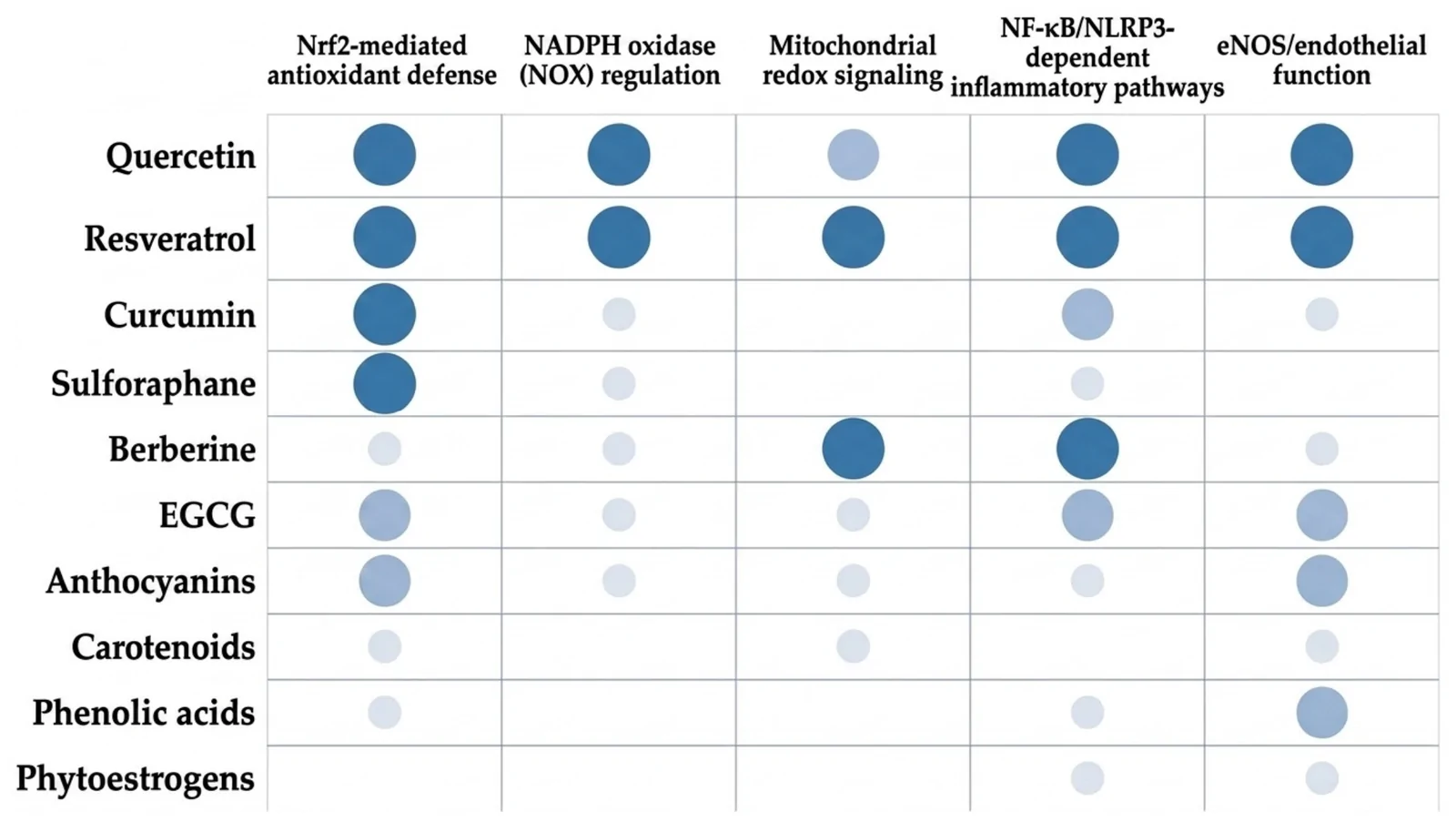
Compound-class evidence maps across the core redox–inflammatory network, distinguishing pathway-dependence evidence (loss-of-function studies) from pathway-associated and supporting evidence for each phytochemical class discussed in Section 4. Created by Urbanowicz T on 24 August 2026 with www.figurelabs.ai (http://www.figurelabs.ai/) (ID: FL-PUB-20260824-NCAQXV).

**Figure 6:**
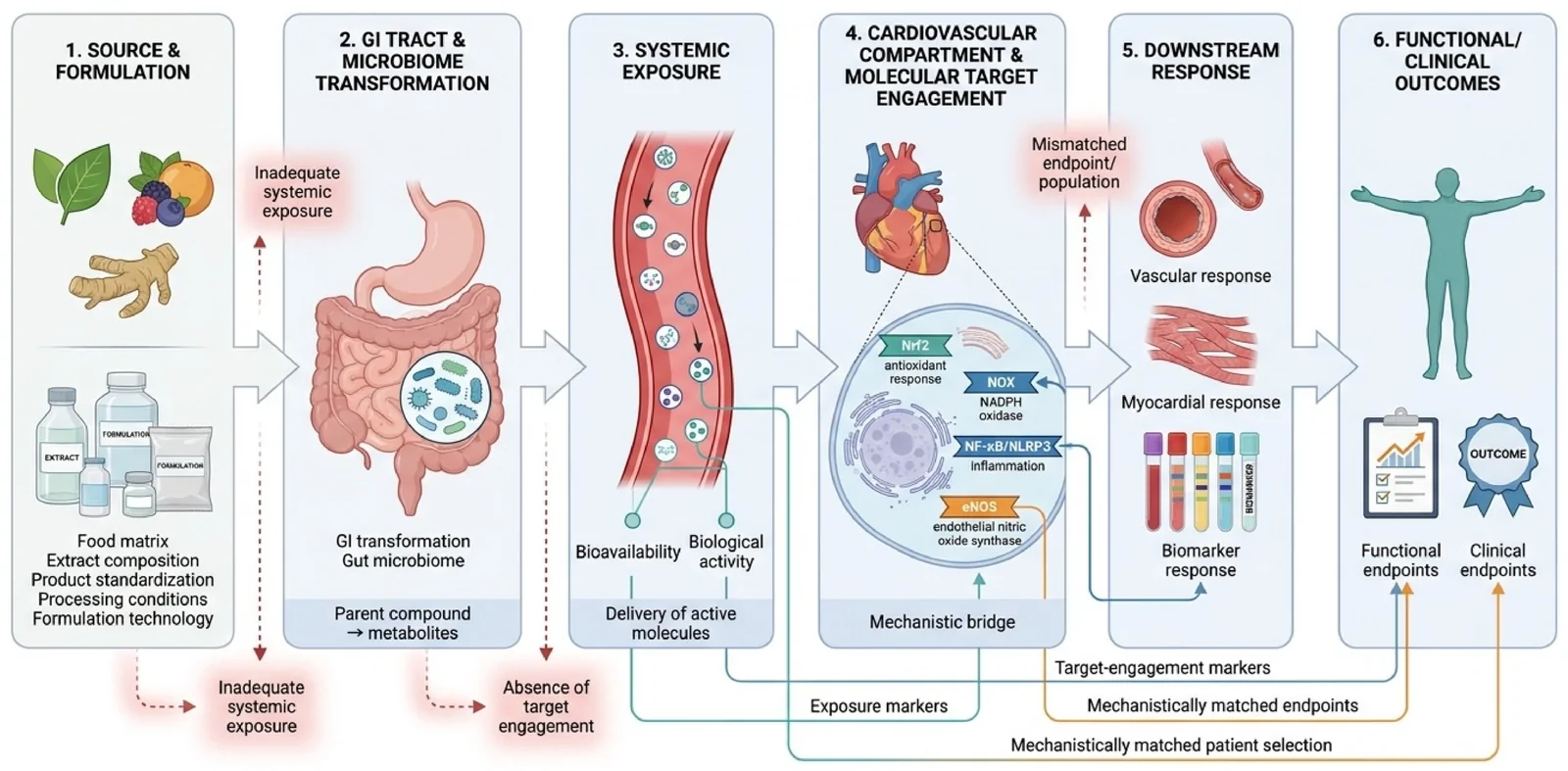
Translational pathway from source and formulation through gut microbial metabotype, systemic exposure, molecular target engagement, and functional or clinical response, to biomarker-guided precision phytotherapy (see Section 6, Section 7 and Section 8). Created by Urbanowicz T on 24 August 2026 with www.figurelabs.ai (http://www.figurelabs.ai/) (ID: FL-PUB-20260824-H91809).

**Table 1:** Molecular mechanisms and strength of target-engagement evidence for representative phytochemicals affecting the cardiovascular redox–inflammatory network.

Compound	Dominant Mechanism	Direct Molecular Target/Interaction	Evidence Level	References
Sulforaphane	Electrophilic target engagement	Keap1 Cys151; other sensor Cys reported	Direct target engagement	[61,66,67,68]
Curcumin	Electrophilic + pathway regulation	Keap1–Nrf2 pathway regulation; direct NLRP3 engagement not established	Pathway/target-associated; pathway-level for NLRP3	[107,176,177,178,179]
Resveratrol	Pathway regulation	Nrf2, NOX, mitochondrial/eNOS signaling; no dominant covalent target established	Pathway dependence/association	[69,84,118,119,157,158,159]
Quercetin	Pathway/enzymatic regulation	Keap1–Nrf2 interaction; NOX regulatory machinery/activity; no demonstrated NLRP3 Cys engagement	Target/pathway-associated	[70,85,86,108]
Berberine	Upstream metabolic regulation	mTOR/mitochondrial ROS–NLRP3 axis; no direct NLRP3 binding established	Pathway-level	[106,203,204,205,206]
Oridonin	Covalent NLRP3 inhibition	NLRP3 Cys279 → impaired NLRP3–NEK7 interaction	Direct target engagement	[221]

Oridonin is included as a mechanistic comparator rather than as a principal cardiovascular phytochemical reviewed in Section 4, because its covalent engagement of NLRP3 provides a well-defined example against which indirect phytochemical modulation of inflammasome signaling can be evaluated.

**Table 2:** Cardiovascular roles of the core redox–inflammatory network nodes.

Node	Principal Cardiovascular Role	Context Dependence	Representative Evidence
Nrf2	Adaptive transcriptional response controlling glutathione, thioredoxin, HO-1, NQO1, metabolism, and stress resistance	Usually protective when appropriately scaled; sustained dysregulation can support maladaptation	[51,60,223]
NOX1/2	Regulated superoxide generation, inflammatory signaling, and endothelial injury	Predominantly pathogenic when chronically activated	[42,43,44]
NOX4	Predominantly hydrogen-peroxide-linked signaling with vascular, myocardial, and renal actions	Protective or detrimental according to localization, abundance, cell type, and disease stage	[42,43,44,45,46]
NOX5	Calcium-responsive ROS source expressed in human cardiovascular tissues	Translationally important because conventional rodents lack the human orthologue	[42,43,44,45,46,80]
NLRP3	Caspase-1 activation, IL-1β/IL-18 maturation, and inflammatory amplification	Pathogenic when persistently activated; activation mechanism depends on initiating damage	[47,87,90,224]
eNOS/NO	Vasodilation and antithrombotic, anti-inflammatory, antiproliferative signaling	Protective when coupled; uncoupled eNOS becomes an ROS source	[26,34,116]
Mitochondria	Bioenergetics, ROS signaling, calcium handling, danger signals, and cell death	Physiological signaling becomes pathological when sustained or amplified	[27,32,39,40]

Abbreviations: eNOS, endothelial nitric oxide synthase; HO-1, heme oxygenase-1; IL, interleukin; NLRP3, NOD-, LRR- and pyrin domain-containing protein 3; NO, nitric oxide; NOX, NADPH oxidase; NQO1, NAD(P)H quinone dehydrogenase 1; Nrf2, nuclear factor erythroid 2-related factor 2; ROS, reactive oxygen species.

**Table 3:** Representative phytochemical classes mapped to mechanisms and translational constraints.

Class/Examples	Mechanistic Emphasis	Human Evidence Signal	Principal Limitation	Refs.
Flavonols/quercetin	NOX, Nrf2, endothelial and metabolic signaling	Modest blood-pressure or risk-factor effects; endothelial findings heterogeneous	Conjugation, food matrix, variable exposure	[121,133,225]
Flavan-3-ols/EGCG	Mitochondrial, NF-κB, Nrf2, endothelial signaling	Vascular effects reported for tea, cocoa, and purified flavan-3-ols	Product composition and caffeine/food-matrix confounding	[139,141,142]
Anthocyanins	Endothelial signaling, lipid oxidation, inflammation, microbiome interactions	Vascular and cardiometabolic effects in selected trials and cohorts	Source, dose, metabolite and baseline-risk heterogeneity	[147,149,151]
Stilbenes/resveratrol	SIRT1-AMPK, Nrf2, mitochondrial and eNOS signaling	FMD and inflammatory signals in selected populations	Low parent exposure and formulation heterogeneity	[157,163,226]
Curcuminoids	Nrf2, NF-κB, mitochondrial stress, inflammasome-permissive conditions	Selected blood-pressure, endothelial, and risk-factor signals	Very low native exposure; formulations not equivalent	[176,177,183]
Organosulfur/sulforaphane	Keap1 cysteine sensing and Nrf2-dependent adaptation	Mechanistic human studies; limited cardiovascular endpoint data	Myrosinase, processing, precursor conversion	[66,67,196]
Alkaloids/berberine	AMPK, metabolism, mitochondrial stress, PCSK9-related and inflammatory pathways	Cardiometabolic risk-factor effects; direct vascular target engagement limited	Low oral exposure, intestinal actions, interaction potential	[203,204,209]
Carotenoids/lycopene, astaxanthin	Membrane and lipoprotein redox environment	Selected lipid, blood-pressure, and oxidative-marker effects	Carrier, formulation, baseline status, tissue distribution	[184,187,189]
Phenolic acids/hydroxytyrosol	LDL oxidation, platelet and endothelial biology	Olive-derived and postprandial vascular evidence	Rapid conjugation and metabolite-dependent activity	[171,173,174]

Abbreviations: AMPK, AMP-activated protein kinase; EGCG, epigallocatechin gallate; eNOS, endothelial nitric oxide synthase; FMD, flow-mediated dilation; Keap1, Kelch-like ECH-associated protein 1; LDL, low-density lipoprotein; NF-κB, nuclear factor kappa B; NOX, NADPH oxidase; Nrf2, nuclear factor erythroid 2-related factor 2; PCSK9, proprotein convertase subtilisin/kexin type 9; SIRT1, sirtuin 1.

**Table 4:** Human evidence and pharmacokinetic interpretation.

Intervention	Human Evidence Considered	Interpretation	Key Caution	References
Resveratrol	Endothelial, inflammatory, and cardiometabolic trials and syntheses	Possible functional vascular benefit in selected populations	Parent exposure is low; doses and formulations vary	[157,160,163,164,226]
Curcumin/turmeric	Endothelial, blood-pressure, and cardiometabolic outcomes	Signals are formulation-dependent and not uniform	Native and enhanced products must not be pooled uncritically	[176,177,178,179,183]
Quercetin	Blood pressure, endothelial function, lipids, and inflammatory markers	Modest effects on selected risk factors	Glycoside, source, background diet, and baseline risk differ	[121,133,134,135,225]
Anthocyanin-rich foods/extracts	Vascular function and cardiometabolic risk markers	Generally favorable but heterogeneous	Metabolite exposure and food matrix often underreported	[147,149,150,151,154]
Lycopene/tomato products	Blood pressure, lipids, oxidative markers, epidemiology	Supportive risk-factor evidence, not outcome proof	Food and isolated supplement interventions differ	[184,185,187,188,189]
Berberine	Glucose, lipids, blood pressure, and composite risk factors	Cardiometabolic activity; cardiovascular outcome evidence insufficient	Product quality, interactions, and gastrointestinal effects	[203,204,209,210,211]

**Table 5:** Translation barriers and precision-trial priorities.

Barrier	Consequence	Recommended Response	Refs.
In vitro concentrations exceed human exposure	Mechanistic effects may not be pharmacologically reachable	Measure parent and active metabolite exposure before efficacy claims	[15,128,166,180,303,306]
Food matrix, processing, and formulation vary	Nominally similar interventions are not equivalent	Specify source, composition, stability, dosing conditions, and formulation	[127,128,155,166,180]
Microbiome-dependent biotransformation	Identical intake produces different active metabolites	Measure metabolites and consider producer/non-producer metabotypes	[127,219,290,297,299,318]
Heterogeneous cardiovascular phenotypes	Average effects dilute pathway-matched responses	Use biomarker-defined enrichment and phenotype-specific endpoints	[300,316]
Drug–phytochemical interactions	Exposure, bleeding, blood pressure, or toxicity may change	Prospectively assess transporters, CYP pathways, antithrombotic and hemodynamic overlap	[212,213,319]
Surrogate endpoints without target engagement	Clinical signal cannot be assigned to the proposed mechanism	Link exposure to pathway biomarkers, function, and then clinical outcomes	[126,129,305]

Abbreviations: CYP, cytochrome P450.

## Data Availability

No new data were created or analyzed in this study. Data sharing is not applicable to this article.

## Abbreviations

ADP, adenosine diphosphate; AMPK, AMP-activated protein kinase; ARE, antioxidant response element; ASC, apoptosis-associated speck-like protein containing a CARD; ATP, adenosine triphosphate; BH_4_, tetrahydrobiopterin; CANTOS, Canakinumab Anti-inflammatory Thrombosis Outcomes Study; COLCOT, Colchicine Cardiovascular Outcomes Trial; Cul3, Cullin-3; Cys, cysteine; e^−^, electron; EGCG, epigallocatechin-3-gallate; eNOS, endothelial nitric oxide synthase; FB3, fructose-bisphosphatase 3; FMD, flow-mediated dilation; GI, gastrointestinal; GSH, glutathione; GST, glutathione S-transferase; HO-1, heme oxygenase-1; IKKβ, inhibitor of nuclear factor κB kinase subunit beta; IL, interleukin; IL-1β, interleukin-1 beta; IL-18, interleukin-18; K^+^, potassium ion; Keap1, Kelch-like ECH-associated protein 1; LDL, low-density lipoprotein; LoDoCo2, Low-Dose Colchicine 2 trial; MCC950, selective NLRP3 inflammasome inhibitor; mRNA, messenger RNA; mTOR, mechanistic target of rapamycin; mtDNA, mitochondrial DNA; mtROS, mitochondrial reactive oxygen species; MyD88, myeloid differentiation primary response protein 88; NADPH, reduced nicotinamide adenine dinucleotide phosphate; NEK7, NIMA-related kinase 7; NF-κB, nuclear factor kappa B; NLRP3, NOD-like receptor family pyrin domain-containing 3; NO, nitric oxide; NOX, NADPH oxidase; NQO1, NAD(P)H quinone oxidoreductase 1; Nrf2, nuclear factor erythroid 2-related factor 2; O_2_•^−^, superoxide anion radical; ONOO^−^, peroxynitrite; P2X7, purinergic receptor P2X7; PCSK9, proprotein convertase subtilisin/kexin type 9; PFK, phosphofructokinase; PKR, pyruvate kinase reaction; RNA Pol II, RNA polymerase II; ROS, reactive oxygen species; SIRT1, sirtuin 1; TLR4, Toll-like receptor 4; TRX, thioredoxin; Ub, ubiquitin.
